# EMG pattern recognition compared to foot control of the DEKA Arm

**DOI:** 10.1371/journal.pone.0204854

**Published:** 2018-10-18

**Authors:** Linda J. Resnik, Frantzy Acluche, Matthew Borgia, Jill Cancio, Gail Latlief, Samuel Phillips, Nicole Sasson

**Affiliations:** 1 Research Department, Providence VA Medical Center, Providence, Rhode Island, United States of America; 2 Health Services, Policy and Practice, Brown University, Providence, Rhode Island, United States of America; 3 Center for the Intrepid, Brooke Army Medical Center JBSA-Ft. Sam Houston, Fort Sam Houston, TX, United States of America; 4 Extremtiy Trauma and Amputation Center of Excellence, Fort Sam Houston, Texas, United States of America; 5 Department of PM&R James A Haley Veteran’s Hospital, Tampa, FL, United States of America; 6 NYU School of Medicine, New York, New York, United States of America; 7 VA NY Health Harbor System, New York, New York, United States of America; TNO, NETHERLANDS

## Abstract

**Introduction:**

EMG pattern recognition control (EMG-PR) is a promising option for control of upper limb prostheses with multiple degrees of freedom (DOF). The purposes of this study were to 1) evaluate outcomes of EMG-PR and inertial measurement units (IMU) control of the DEKA Arm as compared to personal prosthesis; and 2) compare outcomes of EMG-PR to IMU control of DEKA Arm.

**Methods:**

This was a quasi-experimental, multi-site study with repeated measures that compared non-randomized groups using two types of controls: EMG-PR and IMUs. Subjects (N = 36) were transradial (TR) and transhumeral (TH) amputees. Outcomes were collected at Baseline (using personal prosthesis), and after in-laboratory training (Part A), and home use (Part B). Data was compared to personal prosthesis, stratified by amputation level and control type. Outcomes were also compared by control type.

**Results:**

The EMG-PR group had greater prosthesis use after Part A, but worse dexterity, lower satisfaction, and slower activity performance compared to Baseline; the IMU group had slower activity performance. After Part B, the EMG-PR group had less perceived activity difficulty; the IMU group had improved activity performance, improved disability and activity difficulty, but slower performance. No differences were observed for TH group by control type in Part A or B. The TR group using EMG-PR had worse dexterity (Parts A & B), and activity performance (Part A) as compared to IMU users.

**Discussion/Conclusion:**

Findings suggest that for the TR group that IMUs are a more effective control method for the DEKA Arm as compared to the EMG-PR prototypes employed in this study. Further research is needed to refine the EMG-PR systems for multi-DOF devices. Future studies should include a larger sample of TH amputees.

**Trial registration:**

ClinicalTrials.gov NCT01551420.

## Introduction

The human arm and hand, with more than 20 degrees of freedom (DOF) is extremely dexterous, exquisitely sensitive, and adept in performing physical and self-care, as well as communication activities. Complete replacement of a missing limb through prosthetic substitution is inherently challenging, given the myriad functions of the upper limb. One inherent challenge is restoration of multiple DOF of the limb, which allows the end effector (or hand) to be used within a wide sphere of movement. [1]

New mechatronic prostheses, like the DEKA Arm (described below), provide increased powered DOF as compared to conventional myoelectric devices, [2] and as such hold great promise. However the control of such multiple powered DOFs remains challenging. Historically, powered upper limb prostheses have been controlled primarily through the use of surface electromyography (sEMG). These sEMG controlled devices, either single site or dual site, are operated by non-physiological and non-intuitive muscle contractions, which are configured to operate specific functions of the prosthetic limb. With higher DOF devices, sEMG utilizes a mode switching function which enables the user to utilize each control site for a second function. This control strategy, while commonly used, may have a higher cognitive burden, is slow, may lack reliability, and be subject to change over time, with fatigue, sweat, and residual limb volume. [2] Thus, it is not surprising that a recent systematic review of the literature found no clear benefit in myoelectric controlled upper limb prosthesis over body-powered upper limb prosthesis.[3] Furthermore, conventional, dual site sEMG control with mode switching can only control two DOF, or at most three DOF, and thus would not be adequate to control the DEKA Arm.

A new control interface method, Inertial Measurement Units (IMUs), attached to the top of a user’s shoes, (described in more detail below) was developed for use with the DEKA Arm. IMU control has proven useful for dozens of research subjects. [4] There are some limitations in the utilization of IMU for upper limb prosthetic control. IMUs cannot be operated by persons with substantial lower limb mobility or balance deficits. Also, the upper limb prosthesis cannot be actively controlled by IMUs when the user is moving his/her feet for activities such as ambulation, and switch to a “standby” mode to protect the user from unintentional arm movements. Finally, this control method is not intuitive, and some users have reported difficulty in substituting foot movements for upper limb movements. Thus, this method of control may not be ideal for persons with executive function deficits. An alternative control method for the DEKA Arm might overcome these limitations.

EMG pattern recognition control (EMG-PR) is a promising control alternative. EMG-PR maps physiologically appropriate muscle contractions made by the user as he/she imagines moving the phantom limb to corresponding movements of the prosthesis, making it potentially more intuitive than direct sEMG or IMU control. Several clinical studies have been published on EMG-PR, but these studies involve prostheses that have only 2 [5] or 3 DOF.[6] No prior study has evaluated the use of EMG-PR in the DEKA Arm, or compared it to IMU control. The purposes of this study were to 1) evaluate outcomes of EMG-PR control and IMU control of the DEKA Arm as compared to personal prosthesis; and 2) compare outcomes of EMG-PR control to IMU control of DEKA Arm. We hypothesized that the EMG-PR would offer distinct advantages over IMU control.

## Methods

### The DEKA Arm

The DEKA Arm is a multi-degree of freedom (DOF) upper limb prosthesis, developed through DARPA’s Revolutionizing Prosthetics Program.[7] It is available in three configuration levels: a radial configuration (RC) for persons with a transradial (TR) amputation; a humeral configuration (HC) for persons with a transhumeral (TH) amputation; and a shoulder configuration (SC) for persons with a shoulder disarticulation, forequarter or very short TH amputation. Complete descriptions of the DEKA Arm and its features have been reported elsewhere.[8] Only the RC and HC DEKA Arms were used in the sub-analyses reported in this manuscript. Both these configuration levels have six hand grip patterns, powered wrist flexion/extension (combined with ulnar and radial deviation) and wrist pronation/supination. The HC DEKA Arm also has powered elbow flexion/extension and humeral internal/external rotation which has an axis of movement several inches proximal to the elbow joint. The RC operates in the hand mode to control grip open/close and wrist movement. The HC operates in both the hand mode and in arm mode, for control of elbow movements and humeral rotation. Both configurations have a standby mode that deactivates DEKA functions and light-emitting diode (LED) wrist displays which provide notification of the grip pattern selected as well as the mode of operation. [8]

#### Control of the DEKA Arm

In this study, the DEKA Arm was controlled by two methods. The first was primary through foot controls using IMUs [4], which were sometimes supplemented with pneumatic bladders, linear transducers and direct control using surface electromyography (sEMG) as desired by the user. This method is referred to throughout this paper as IMU. IMUs are secured to the top of the shoe with a clip. Users move their feet/ankles in pre-programmed directions to operate DEKA functions (dorsiflexion, plantarflexion, eversion and inversion). IMUs automatically detect walking motions and include a walk detect feature that reduce the likelihood of unintentionally activating DEKA functions. IMU use is limited to persons who are not missing both lower limbs or have impaired lower limb function.

The second control method used in this study was sEMG Pattern Recognition control (EMG-PR).The EMG-PR control used in this study was developed by Coapt LLC (Chicago, Ill) in collaboration with DEKA Integrated Solutions (Manchester, NH). Two prototypes of the system were utilized in this study. Both use 8 dome electrode pairs and 1 reference electrode placed on the residual limb to register patterns of muscle contractions. Each distinct pattern is assigned to operate prosthesis functions through a standardized calibration process.

Prototype 1 could be used with 8 distinct patterns of muscle contractions and a “mode switching” function- which enabled the user to control up to 4 functions: 4 powered DOFs, or 3 DOFs and the grip selection function (to choose between six different grip patterns). Prototype 1 interfaced with the DEKA Arm using a multi-connection interface cable (i.e. mating cable) which was connected to an Arm Control Unit (ACI), mounted on the prosthetic socket. Pressure transducers connected to the ACI were used to operate DEKA functions, including mode switching and grip selection as needed.

Prototype 2 could be used with up to 12 distinct patterns of muscle contractions to operate up to six functions of the DEKA Arm, and thus did not require mode switching to move between arm and hand modes. This prototype was connected to the DEKA Arm though a CAN Bus connection cable and ACIs were used to connect pressure transducers as needed.

### Study design

This was a quasi-experimental, multi-site study with a repeated measures design. IRBs at each participating site (Center for the Intrepid at Brooke Army Medical Center (CFI), VA New York Harbor HealthCare System, the James A. Haley VAMC) and the Providence VA Medical Center approved the study protocol. The sample included in this analysis was a subgroup of subjects in the larger VA Home Study of the DEKA Arm who participated in the study between 6/11/2012 and12/19/2017. Consented subjects were fit and trained with a DEKA Arm controlled either by EMG-PR or by IMUs and other controls. Data was collected at Baseline, with the existing prosthesis (for prosthesis users), and at repeated intervals. The study had two components, in-laboratory training (Part A), and home use (Part B).

#### Sample

The sample was a convenience sample, with sample size largely determined by availability of funding. Subjects were eligible to participate in Part A of the full study if they were: (a) 18 years and older; (b) had an amputation at the transradial (TR), tranhumeral (TH), or shoulder disarticulation or scapulothoracic (SD) level; (c) had no health conditions that might limit their participation; and (d) were able to wear a prosthesis. Subjects were recruited by local site clinicians, flyers and brochures, through advertisements, listservs and press releases. The sub-analysis presented in this manuscript includes only those subjects with TR or TH amputation who used either a RC or HC DEKA Arm. Subjects who had completed Part A were eligible to enroll in Part B if they demonstrated: (a) adequate performance with the DEKA prosthesis while utilizing safety awareness and (b) were able to troubleshoot minor technical problems with minimal guidance. To increase compliance, subjects were provided with modest financial incentives (non-active duty only) and reimbursement for travel expenses as needed. Allocation of subjects to intervention groups was performed sequentially. Initially, all subjects were provided with IMU controls (allocated to the IMU control group). When funding to study EMG-PR was obtained, all later subjects were provided with EMG-PR controls (allocated to EMG-PR control group).

#### Prosthetic training

Individualized prosthetic training was led by the study occupational therapist (OT) at each site. During training, subjects learned to don and doff the DEKA Arm, and to understand and operate its features. During Virtual Reality Environment (VRE) training, IMU users were oriented to DEKA Arm operations using a standardized protocol [9, 10]. EMG-PR users utilized the virtual reality system provided by the COAPT Complete Control software (COAPT Complete Control, Chicago, IL). EMG-PR users were introduced to the calibration process during this phase. Once subjects demonstrated the ability to control the avatar in the VRE with their respective controls, they progressed to active prosthesis training. Active training began with repetitions of the controls and simple grasp and release activities in each of the grasp patterns. As the subjects improved with these basic tasks, training progressed to more complex functional tasks and everyday activities. Subjects also participated in community outings where they were required to operate the device while shopping, eating a meal in public and using transportation (as a passenger). The training protocol required a minimum of 10 hours of training for users of the RC and HC DEKA Arm. Each training session was typically 2 hours in length, with rest breaks every 30 minutes or as needed. Training continued until the study team agreed that the subject had reached a plateau in terms of skill, until the maximum number of training hours were reached (capped at 40 hours for IMU users), or to avoid the potential for missing data due to drop-out, if the subject expressed a strong desire to end participation due to scheduling conflicts, or personal reasons.

#### Data collection

Prosthetic training and data collection took place at three participating study sites, which were VA or DoD Medical Centers on subject characteristics, prior prosthesis experience and current use of a prosthesis was collected at Baseline. Outcome measures were administered at Baseline at the End of Part A (EOA) and at the End of Part B (EOB).

A suite of validated performance and self-report measures were collected. Performance measures included: modified Jebsen-Taylor Hand Function test (JTHFT) [11, 12]; Activities Measure for Upper Limb Amputees (AM-ULA) [13], University of New Brunswick Test of Prosthetic Function for Unilateral Amputees (UNB) [14], Timed Measure of Activity Performance (T-MAP)[15] and Brief Activity Measure for Upper Limb Amputees (BAM-ULA) [16]. JTHFT, UNB, AM-ULA and BAM-ULA were only collected at Baseline if the subject was a prosthesis user and had a functioning prosthesis at the time of the visit. Self-report measures included: Quick Disabilities of the Arm, Shoulder and Hand Score (QuickDASH) [17]; Upper Extremity Functional Scale (UEFS) [18]; Patient Specific Functional Scale (PSFS) [19]; Wong-Baker FACES Pain Rating Scale (Wong-Baker) [20]; Quality of Life (QOL) scale [21]; Community Reintegration of Service Members Computer Adaptive Test (CRIS-CAT) [22]; and Trinity Amputation and Prosthesis Experience Prosthetic Satisfaction Scale (TAPES).[23] Table 1 provides a synopsis of the content of each of these measures.

**Table 1:** Outcome measures.

Measure	Construct	Brief description	Response	Higher scores indicate…
**Dexterity**				
Jebsen-Taylor Hand Function Test (JTHF)	Dexterity	7 tests of hand function	Performance speed; items .sec	better performance
**Activity**				
Activities Measure for Upper-Limb Amputees (AM-ULA)	Activity performance	18-everyday tasks	Task completion: speed, movement quality, skill and independence	better performance
University of New Brunswick Test of Prosthetic Function (UNB): Skill	Prosthetic skill	10 components of daily tasks requiring bimanual engagement	Skillfulness of terminal device use.	better performance
University of New Brunswick Test of Prosthetic Function (UNB): Spontaneity	Prosthetic spontaneity	10 components of daily tasks requiring bimanual engagement	Spontaneity of engaging the prosthesis in activities	better performance
Timed Measure of Activity Performance (T-MAP)	Activity performance	5 activities of daily living	Task completion: speed	worse performance
Brief Activity Measure for Upper Limb Amputees (BAM-ULA)	Activity performance	10 items of functional task performance	Task completion: Unable to complete; Can complete	better performance
**Self-reported function**				
Disabilities of the Arm, Shoulder and Hand Score (QuickDASH)	Disability	Self-reported functional difficulty (8 items) 3 items about sleep, sensation and pain	Performance difficulty and impairment severity	greater disability
Upper-Extremity Functional Scale (UEFS)	Activity performance	Self-reported difficulty performing 23 everyday activities	Difficulty in performance	greater difficulty
Upper-Extremity Functional Scale (Use)	Use of prosthesis	Self-reported use of the prosthesis during everyday activities	Prosthesis use	more activities done with prosthesis
Patient-Specific Functional Scale (PSFS)	Difficulty performing activities	5 self-selected activities difficult to do because of the amputation	Difficulty in performance	less difficulty
**Other measures**				
Wong-Baker FACES Pain Rating Scale	Pain	Six faces showing levels of pain severity	Pain intensity	more pain
Quality of Life (QOL)	Quality of life	16 question items about quality of life	Satisfaction with quality of life	better QOL
The Community Reintegration of Service Members Computer Adaptive test (CRIS-CAT)		Computer adaptive testing measuring participation in life roles		better community integration
CRIS-CAT Extent of Participation	Extent of participation		Frequency and amount	
CRIS-CAT Perceived Limitations	Perceived difficulty		Perceived limitations	
CRIS-CAT Satisfaction with Participation	Satisfaction		Satisfaction scale	
Trinity Amputation and Prosthesis Experience Scales (TAPES)	Prosthetic satisfaction	10 items satisfaction with prosthesis	Satisfaction	greater satisfaction

#### Data analyses

Descriptive statistics were used to characterize subject characteristics by control type, stratified by amputation level. Scores of outcome measures at Baseline were compared to EOA and EOB for each group (EMG-PR and IMU) using paired Wilcoxon signed-rank tests. Scores of those lost to follow-up or with missing outcomes data were omitted from the analyses. These analyses were performed for the full sample, and repeated after stratifying by amputation level. Effect sizes (ES) were calculated for all estimates. In our clinical trials registration, the primary outcome measure was identified as a change in the QOL scale from baseline to end of the home use period. However, earlier analyses revealed that this measure was not sensitive to change due to the DEKA Arm. Thus, all outcomes were analyzed. Scores of outcome measures at EOA and EOB were compared by control group (EMG-PR and IMU) using Wilcoxon rank-sum tests. Given that there appeared to be age differences between amputation level and control groups, separate linear regression models for outcome measures controlling for age as a continuous covariate were generated. These models were stratified by DEKA Arm configuration level, and results compared to determine if controlling for age changed the significance or directionality of findings. We examined residual vs. predicted and Q-Q plots for outcomes that were statistically significantly different by control group in either the Wilcoxon ranksum or the regression analyses. All analyses were performed in SAS 9.4 (Carey, NC).

## Results

### Sample characteristics

**Fig 1:**
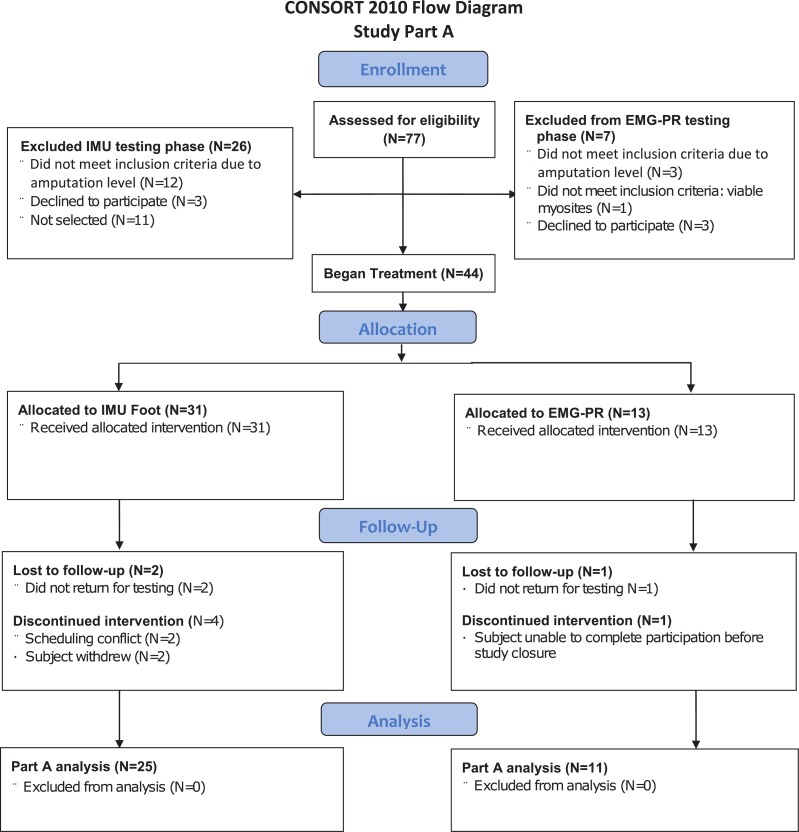
CONSORT 2010 Flow Diagram Part A.

Fig 1 shows the number of subjects screened and allocated to interventions in Part A as well as the reasons for loss to follow-up and discontinuation. Seventy seven subjects completed study screening visits, 31 were allocated to the IMU control phase and 13 to the EMG-PR phase. Six subjects in the IMU and 2 subjects in the EMG-PR groups were lost to follow-up or discontinued during Part A. Thus, the sample for this analysis included 36 subjects fit with a radial or humeral configuration DEKA Arm (Table 2). Among the 11 persons in the EMG-PR group (mean age 44.9 yrs, 82% male), 9 subjects had TR amputation and 2 TH. Among the IMUs group (mean age 43.0, 88% male), 17 had an amputation at the TR level and 8 at the TH level. Subjects in the EMG-PR group had an average of 22.7 (sd 8.0) hours of training and those in the IMU group had 15.8 (sd 6.3) hours, differences between training hours were significantly different for the TR group, but not the TH group (p<0.05).

**Table 2:** Subject demographics by control type and amputation level.

	EMG-PR (N = 11)			Other (N = 25)		
	TR (N = 9)	TH (N = 2)	Total	TR (N = 17)	TH (N = 8)	Total
	Mn (sd)	Mn (sd)	Mn (sd)	Mn (sd)	Mn (sd)	Mn (sd)
**Age**	46.5 (18.3)	32.5 (6.0)	44.9 (17.4)	38.7 (15.0)	52.1 (10.9)	43.0 (15.0)
**Number of training hours**	22.0 (8.9)	25.0 (7.1)	22.5 (8.3)	13.8 (5.6)	20.0 (5.9)	15.8 (6.3)
	**N (%)**	**N (%)**	**N (%)**	**N (%)**	**N (%)**	**N (%)**
**Sex**						
Male	7 (77.9)	2 (100.0)	9 (81.8)	14 (82.4)	8 (100.0)	22 (88.0)
Female	2 (22.2)	0 (0.0)	2 (18.2)	3 (17.7)	0 (0.0)	3 (12.0)
**PR version **						
Initial prototype	3 (33.3)	0 (0.0)	3 (27.3)	NA	NA	NA
Updated prototype	5 (55.66)	0 (0.0)	5 (45.4)	NA	NA	NA
Both prototypes	1 (11.1)	2 (100.0)	3 (27.3)	NA	NA	NA
**Prosthesis user at time of enrollment**						
No	0 (0.0)	1 (50.0)	1 (9.1)	4 (23.5)	0 (0.0)	4 (16.0)
Yes	9 (100.0)	1 (50.0)	10 (90.0)	13 (76.5)	8 (100.0)	21 (84.0)
**Tested with a prosthesis at Baseline**						
No	2 (22.2)	1 (50.0)	3 (27.3)	5 (29.4)	1 (12.5)	6 (24.0)
Yes	7 (77.9)	1 (50.0)	8 (72.7)	12 (70.6)	7 (87.5)	19 (76.0)

**Fig 2:**
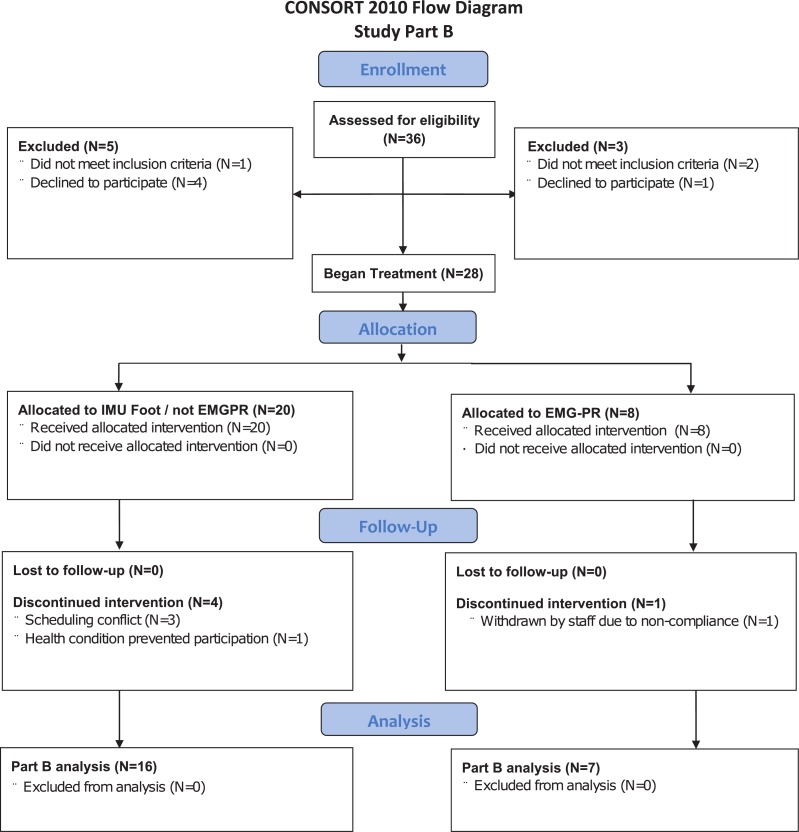
CONSORT 2010 Flow Diagram Part B.

All subjects who completed Part A were evaluated for eligibility in Part B (Fig 2) Twenty of the 36 subjects assessed for eligibility began Part B with IMU controls, and 8 began Part B with EMG-PR controls. Four subjects in the IMU group and 1 in the EMG-PR group discontinued Part B. Thus, the number of subjects analyzed from Part B was 16 from the IMU group and 7 from the EMG-PR group.

### Baseline to EOA comparisons

Comparisons between outcomes measures at Baseline and EOA are shown in Table 3. In the EMG-PR group, several JTHFT subtests scores were lower and T-MAP scores slower at EOA (EOA) compared to Baseline (p<0.05). UEFS scores were higher indicating greater perceived difficulty in activity performance (p = 0.04). In contrast, PSFS scores were significantly higher at EOA (p-0.02). Scores of the TAPES satisfaction were lower for the EMG-PR controlled DEKA Arm as compared to personal prosthesis (p = 0.02).

**Table 3:** Comparing outcomes at Baseline to End of A (EOA) by control type.

	EMG PR (N = 11)					Other (N = 25)				
		Baseline	EOA	W S-R			Baseline	EOA	W S-R	
	N	Mn (sd)	Mn (sd)	P	ES	N	Mn (sd)	Mn (sd)	P	ES
**Dexterity**	** **									
Jebsen-Taylor Hand Function (JTHFT)										
JTHFT: Writing	8	0.38 (0.19)	0.42 (0.13)	0.84	0.25	19	0.37 (0.21)	0.35 (0.15)	0.59	-0.11
JTHFT: Page Turning	8	0.10 (0.03)	0.06 (0.02)	**0.04**	-1.57	19	0.08 (0.07)	0.07 (0.04)	0.82	-0.18
JTHFT: Small items	8	0.06 (0.05)	0.04 (0.04)	0.55	-0.44	19	0.07 (0.08)	0.10 (0.08)	0.38	-0.38
JTHFT: Feeding / Eating	8	0.13 (0.06)	0.05 (0.04)	**0.02**	-1.57	19	0.11 (0.08)	0.08 (0.05)	0.17	-0.45
JTHFT: Checkers	8	0.12 (0.06)	0.05 (0.05)	**0.02**	-1.27	19	0.10 (0.08)	0.09 (0.07)	0.57	-0.12
JTHFT: Light Cans	8	0.21 (0.09)	0.09 (0.08)	**0.02**	-1.41	19	0.21 (0.13)	0.24 (0.16)	0.13	0.21
JTHFT: Heavy Cans	8	0.25 (0.12)	0.11 (0.10)	**0.04**	-1.27	19	0.23 (0.15)	0.24 (0.17)	0.52	0.06
**Activity**										
AM-ULA	8	2.0 (0.3)	1.7 (0.3)	0.17	-0.90	19	17.6 (5.3	18.0 (4.6)	0.78	0.08
UNB: Spontaneity	8	3.1 (0.5)	3.0 (0.5)	0.33	-0.30	18	3.1 (0.4)	3.1 (0.4)	0.99	0.00
UNB: Skill	8	2.9 (0.5)	2.8 (0.6)	0.69	-0.22	18	3.0 (0.5)	3.0 (0.5)	0.84	0.04
T-MAP	9	340.2 (81.8)	549.0 (257.5)	**0.04**	1.09	22	445.9 (216.4)	659.1 (403.4)	**<0.001**	0.66
BAM-ULA	6	7.3 (2.6)	7.5 (2.6)	1.00	0.07	13	7.3 (3.0)	7.8 (1.6)	0.66	0.19
**Self-reported function**										
QuickDASH	11	29.8 (9.7)	27.5 (7.6)	0.64	-0.26	25	30.0 (14.3)	23.7 (1.6)	**0.02**	-0.47
Upper Extremity Functional Scale (UEFS)	8	35.1 (15.8)	46.0 (8.8)	**0.04**	0.85	13	42.6 (5.6)	44.1 (4.9)	0.47	0.29
UEFS use	11	0.4 (0.3)	0.6 (0.3)	0.15	0.63	25	0.4 (0.3)	0.7 (0.4)	**0.00**	0.94
Patient Specific Functional Scale (PSFS)	11	3.5 (2.2)	6.0 (1.9)	**0.02**	1.23	25	3.0 (1.5)	5.4 (1.9)	**<0.0001**	1.39
**Quality of life and Other.**										
Wong-Baker Pain Scale	11	0.7 (1.0)	0.8 (0.8)	0.78	0.10	25	0.8 (1.2)	1.0 (1.1)	0.45	0.14
Quality of Life (QOL) Scale	11	5.3 (0.7)	5.4 (0.7)	0.18	0.16	25	5.9 (0.7)	5.9 (0.7)	0.75	0.07
CRIS-CAT										
Extent of Limitations	11	51.9 (9.3)	52.4 (8.2)	0.73	0.05	24	53.1 (8.4)	54.3 (9.2)	0.30	0.25
Perceived Limitations	11	48.6 (6.4)	49.6 (5.8)	0.88	0.15	24	54.3 (11.0)	54.3 (13.6)	0.63	0.00
Satisfaction w/ Participation	11	47.6 (6.4)	48.3 (4.7)	0.75	0.11	24	53.7 (10.2)	52.2 (6.7)	0.52	-0.17
TAPES Satisfaction Scale	10	3.7 (0.8)	3.0 (0.7)	**0.02**	-0.98	20	3.5 (0.6)	3.6 (0.8)	0.79	0.14

In the IMU group, there were statistically significantly slower T-MAP scores at EOA as compared to Baseline (p<0.001), but lower QuickDASH scores (p = 0.02), and higher UEFS use scores (p = 0.001). Subjects also had higher PSFS scores at the EOA (p<0.0001).

### Baseline to EOB comparisons

In the EMG-PR group, the only statistically significant differences between Baseline and EOB were PSFS scores (p = 0.03) (Table 4). In the IMUs group, there were significantly higher AM-ULA scores (p = 0.001), significantly slower T-MAP scores (p = 0.05), lower (better) QuickDASH scores (p = 0.05), higher UEFS use scores (p = 0.01), higher PSFS scores (p<0.0001), and greater pain on the Wong-Baker Pain Scale (p = 0.02).

**Table 4:** Comparison of outcomes at Baseline and End of B (EOB) by control type.

	EMG PR (N = 7)					Other (N = 16)				
		Baseline	EOB	W S-R			Baseline	EOB	W S-R	
	N	Mn (sd)	Mn (sd)	P	ES	N	Mn (sd)	Mn (sd)	P	ES
**Dexterity**										
Jebsen-Taylor Hand Function (JTHFT)										
JTHFT: Writing	5	0.40 (0.18)	0.46 (0.18)	0.13	0.33	13	0.39 (0.23)	0.46 (0.21)	0.38	0.32
JTHFT: Page Turning	5	0.09 (0.04)	0.08 (0.03)	0.63	-0.28	13	0.10 (0.07)	0.11 (0.07)	1.00	0.14
JTHFT: Small items	5	0.06 (0.05)	0.03 (0.03)	0.44	-0.73	13	0.08 (0.09)	0.09 (0.07)	0.59	0.12
JTHFT: Feeding / Eating	5	0.16 (0.05)	0.10 (0.03)	0.13	-1.46	13	0.12 (0.08)	0.08 (0.08)	0.11	-0.50
JTHFT: Checkers	5	0.11 (0.07)	0.5 (0.04)	0.06	-1.05	13	0.09 (0.08)	0.12 (0.08)	0.11	0.38
JTHFT: Light Cans	5	0.21 (0.08)	0.12 (0.06)	0.13	-1.27	13	0.22 (0.14)	0.27 (0.18)	0.17	0.31
JTHFT: Heavy Cans	5	0.25 (0.14)	0.18 (0.08)	0.63	-0.61	13	0.25 (0.15)	0.30 (0.16)	0.31	0.32
**Activity**										
AM-ULA	5	1.9 (0.4)	2.0 (0.2)	0.81	0.22	12	16.3 (5.3)	20.2 (4.5)	**<0.001**	0.80
UNB: Spontaneity	5	3.0 (0.6)	3.5 (0.3)	0.13	0.91	12	3.1 (0.4)	3.3 (0.4)	0.19	0.53
UNB: Skill	5	2.8 (0.7)	3.2 (0.4)	0.13	0.83	12	3.0 (0.5)	3.2 (0.4)	0.11	0.47
T-MAP	6	335.0 (96.2)	443.3 (154.2)	0.09	0.84	14	449.4 (260.4)	624.8 (429.1)	**0.05**	0.49
BAM-ULA summary (new)	3	6.7 (3.5)	8.7 (1.5)	1.00	0.74	10	7.7 (2.2)	8.3 (1.5)	0.66	0.32
**Self-reported function**										
QuickDASH	7	28.3 (7.3)	27.6 (9.4)	1.00	-0.08	16	26.0 (11.1)	19.7 (10.8)	**0.05**	-0.57
Upper Extremity Functional Scale (UEFS)	5	33.9 (19.7)	41.5 (8.3)	0.81	0.50	9	42.6 (5.6)	38.2 (9.8)	0.36	-0.55
UEFS use	7	0.3 (0.3)	0.1 (0.1)	0.19	-0.88	16	0.4 (0.3)	0.6 (0.2)	**0.01**	0.93
Patient Specific Functional Scale (PSFS)	7	3.4 (2.7)	6.1 (1.1)	**0.03**	1.33	16	2.9 (1.2)	6.8 (1.9)	**<0.0001**	2.42
**Quality of life etc.**										
Wong-Baker Pain Scale	7	0.4 (0.8)	0.6 (0.8)	1.00	0.18	16	0.4 (0.7)	0.9 (1.0)	**0.02**	0.57
Quality of Life (QOL) Scale	7	5.3 (0.8)	5.2 (0.9)	0.95	-0.13	16	5.9 (0.6)	6.0 (0.7)	0.35	0.16
CRIS-CAT										
Extent of Limitations	7	53.0 (10.1)	51.3 (8.6)	0.66	-0.18	16	54.4 (7.8)	58.8 (8.4)	0.06	0.54
Perceived Limitations	7	49.7 (5.9)	47.6 (4.6)	0.41	-0.40	16	55.9 (12.2)	58.6 (15.0)	0.55	0.20
Satisfaction w/ Participation	7	49.1 (7.4)	48.7 (5.7)	0.88	-0.07	16	55.3 (11.1)	54.6 (9.3)	0.84	-0.06
TAPES Satisfaction Scale	6	3.7 (0.9)	2.8 (1.1)	0.31	-0.85	14	3.5 (0.5)	3.8 (1.0)	0.29	0.34

### Direct comparisons between EMG-PR and IMUs group

At EOA, the TR subgroup using EMG-PR had significantly worse scores in JTHFT Checkers, Light Cans, and Heavy Cans (all p<0.05), and worse AM-ULA scores (p = 0.01) (Table 5). Subjects with TH amputation in the EMG-PR group had worse QuickDASH scores (p = 0.05) and lower TAPES satisfaction (P = 0.03) as compared to the IMU group (Table 6).

**Table 5:** Comparing outcomes by control type at End of A / End of B (TR only).

	End of A (N = 27)						End of B (N = 15)					
	EMG-PR (N = 9)		Other (N = 17)		W R-S		EMG-PR (N = 5)		Other (N = 10)		W R-S	
	N	Mn (sd)	N	Mn (sd)	P	ES	N	Mn (sd)	N	Mn (sd)	P	ES
**Dexterity**												
Jebsen-Taylor Hand Function (JTHFT)												
JTHFT: Writing	9	0.41 (0.13)	17	0.44 (0.18)	0.63	0.18	5	0.41 (0.19)	10	0.55 (0.15)	**0.04**	0.86
JTHFT: Page Turning	9	0.06 (0.03)	17	0.07 (0.03)	0.22	0.33	5	0.08 (0.03)	10	0.13 (0.08)	0.08	0.73
JTHFT: Small items	9	0.04 (0.04)	17	0.10 (0.09)	0.07	0.78	5	0.05 (0.05)	10	0.10 (0.07)	0.12	0.78
JTHFT: Feeding / Eating	9	0.05 (0.05)	17	0.09 (0.06)	0.06	0.70	5	0.11 (0.04)	10	0.11 (0.08)	0.93	0.00
JTHFT: Checkers	9	0.05 (0.05)	17	0.11 (0.06)	**0.02**	1.06	5	0.04 (0.03)	10	0.13 (0.08)	**0.01**	1.31
JTHFT: Light Cans	9	0.10 (0.08)	17	0.31 (0.11)	**<0.001**	2.08	5	0.14 (0.07)	10	0.33 (0.16)	**0.01**	1.37
JTHFT: Heavy Cans	9	0.13 (0.10)	17	0.33 (0.11)	**<0.001**	1.87	5	0.17 (0.09)	10	0.36 (0.12)	**<0.01**	1.70
**Activity**												
AM-ULA	9	1.6 (0.3)	17	2.0 (0.4)	**0.01**	1.05	5	2.0 (0.2)	9	2.2 (0.4)	0.16	0.62
UNB: Spontaneity	9	3.0 (0.5)	16	3.2 (0.5)	0.41	0.36	5	3.4 (0.3)	9	3.4 (0.3)	1.00	-0.03
UNB: Skill	9	2.8 (0.6)	16	3.0 (0.6)	0.46	0.31	5	3.2 (0.4)	9	3.2 (0.4)	0.82	0.17
T-MAP	7	494.4 (264.1)	14	598.2 (475.2)	0.80	0.25	4	377.8 (48.8)	9	511.1 (392.3)	0.85	0.40
BAM-ULA summary (new)	8	6.9 (2.2)	12	8.3 (1.4)	0.14	0.77	4	8.5 (1.3)	9	8.7 (1.0)	0.98	0.16
**Self-reported function**												
QuickDASH	9	27.3 (8.3)	17	21.3 (14.4)	**0.05**	-0.47	5	28.6 (10.9)	10	15.0 (8.8)	**0.03**	-1.44
Upper Extremity Functional Scale (UEFS)	7	46.2 (9.2)	14	44.2 (7.3)	0.48	-0.24	3	47.5 (2.3)	8	32.8 (9.5)	**0.01**	-1.74
UEFS use	9	0.5 (0.3)	17	0.6 (0.4)	0.50	0.22	5	0.0 (0.0)	10	0.6 (0.2)	**<0.001**	4.13
Patient Specific Functional Scale (PSFS)	9	5.8 (2.0)	17	5.4 (1.9)	0.78	-0.17	5	5.9 (1.3)	10	7.0 (2.1)	0.13	0.61
**Quality of life etc.**												
Wong-Baker Pain Scale	9	0.8 (0.7)	17	0.8 (1.2)	0.62	0.04	5	0.6 (0.9)	10	0.6 (0.7)	1.00	0.00
Quality of Life (QOL) Scale	9	5.5 (0.5)	17	5.8 (0.8)	0.42	0.40	5	5.2 (0.8)	10	6.0 (0.7)	0.09	1.07
Community integration CRIS-CAT												
Extent of Limitations	9	53.9 (5.6)	17	55.7 (10.6)	0.61	0.19	5	52.2 (8.1)	10	60.1 (9.4)	0.13	0.87
Perceived Limitations	9	49.6 (5.3)	17	54.8 (15.4)	0.62	0.40	5	47.6 (5.5)	10	59.2 (15.9)	0.10	0.85
Satisfaction with Participation	9	48.3 (3.9)	17	51.4 (6.6)	0.27	0.53	5	47.4 (6.0)	10	53.6 (8.7)	0.22	0.78
TAPES Satisfaction Scale	9	2.9 (0.6)	17	3.5 (0.7)	**0.03**	0.92	5	2.5 (0.8)	10	3.8 (1.1)	**0.02**	1.34

**Table 6:** Comparing outcomes by control type at End of A / End of B (TH only).

	End of A (N = 14)						End of B (N = 8)					
	EMG-PR (N = 2)		Other (N = 8)		W R-S		EMG-PR (N = 2)		Other (N = 6)		W R-S	
	N	Mn (sd)	N	Mn (sd)	P	ES	N	Mn (sd)	N	Mn (sd)	P	ES
**Dexterity**												
Jebsen-Taylor Hand Function (JTHFT)												
JTHFT: Writing	2	0.33 (0.10)	8	0.27 (0.11)	0.40	-0.55	2	0.43 (0.15)	6	0.30 (0.15)	0.43	-0.87
JTHFT: Page Turning	2	0.07 (0.02)	8	0.04 (0.02)	0.09	-1.50	2	0.06 (0.02)	6	0.07 (0.04)	0.79	0.27
JTHFT: Small items	2	0.04 (0.02)	8	0.05 (0.02)	0.40	0.50	2	0.09 (0.09)	6	0.06 (0.03)	0.86	-0.65
JTHFT: Feeding / Eating	2	0.09 (0.04)	8	0.07 (0.05)	1.00	-0.41	2	0.09 (0.00)	6	0.06 (0.06)	0.29	-0.55
JTHFT: Checkers	2	0.04 (0.02)	8	0.04 (0.04)	0.89	0.00	2	0.09 (0.04)	6	0.06 (0.02)	0.14	-1.22
JTHFT: Light Cans	2	0.08 (0.02)	8	0.08 (0.06)	0.53	0.00	2	0.13 (0.04)	6	0.13 (0.05)	0.86	0.00
JTHFT: Heavy Cans	2	0.13 (0.07)	8	0.09 (0.05)	0.53	-0.76	2	0.13 (0.01)	6	0.16 (0.05)	0.43	0.65
**Activity**												
AM-ULA	2	1.9 (0.4)	8	14.5 (1.8)	0.07	-2.24	2	1.9 (0.0)	6	1.7 (0.2)	0.29	-1.11
UNB: Spontaneity	2	3.5 (0.1)	8	2.9 (0.3)	0.07	-1.94	2	3.5 (0.4)	6	3.1 (0.5)	0.32	-0.83
UNB: Skill	2	3.3 (0.1)	8	2.7 (0.3)	0.07	-2.21	2	3.3 (0.1)	6	3.0 (0.5)	0.71	-0.75
T-MAP	2	740.0 (134.4)	8	765.6 (220.1)	0.89	0.12	2	574.5 (245.4)	5	829.4 (457.2)	0.57	0.60
BAM-ULA summary (new)	2	8.5 (0.7)	8	6.5 (1.1)	0.14	-2.00	1	8.0 (.)	4	7.8 (2.1)	1.00	NA
**Self-reported function**												
QuickDASH	2	28.4 (4.8)	8	28.7 (6.7)	0.98	0.04	2	25.0 (6.4)	6	27.7 (9.6)	0.61	0.29
Upper Extremity Functional Scale (UEFS)	2	44.5 (5.3)	8	43.7 (4.4)	1.00	-0.18	2	32.6 (0.8)	5	45.4 (2.8)	0.10	5.03
UEFS use	2	0.7 (0.5)	8	0.7 (0.3)	1.00	0.15	2	0.2 (0.3)	6	0.6 (0.2)	0.18	2.11
Patient Specific Functional Scale (PSFS)	2	7.2 (0.5)	8	5.3 (2.1)	0.27	-0.93	2	6.8 (0.3)	6	6.4 (1.7)	1.00	-0.24
**Quality of life etc.**												
Wong-Baker Pain Scale	2	1.0 (1.4)	8	1.3 (0.7)	1.00	0.30	2	0.5 (0.7)	6	1.5 (1.2)	0.64	0.87
Quality of Life (QOL) Scale	2	5.1 (1.3)	8	6.2 (0.6)	0.38	1.56	2	5.2 (1.5)	6	5.9 (0.7)	0.57	0.89
Community integration CRIS-CAT												
Extent of Limitations	2	45.5 (17.7)	8	56.0 (6.1)	0.51	1.24	2	49.0 (12.7)	6	56.5 (6.7)	0.43	0.94
Perceived Limitations	2	49.5 (10.6)	8	54.9 (9.1)	0.42	0.58	2	47.5 (2.1)	6	57.7 (14.8)	0.39	0.75
Satisfaction with Participation	2	48.0 (9.9)	8	56.5 (9.4)	0.44	0.89	2	52.0 (4.2)	6	56.3 (10.9)	0.86	0.43
TAPES Satisfaction Scale	2	3.5 (0.6)	8	3.9 (0.9)	0.49	0.41	2	3.3 (1.9)	6	4.0 (0.8)	0.64	0.73

At EOB, the TR subgroup using EMG-PR had significantly worse JTHFT Writing, Checkers, Light Cans, and Heavy Cans scores (all p<0.05) as compared to those using IMUs. They also had worse QuickDASH (p = 0.03), UEFS scores (p = 0.01), UEFS use scores (p<0.001) and TAPES satisfaction scores (p = 0.02) (Table 5). There were no statistically significant differences between EMG-PR and IMU groups for subjects with TH amputation at either EOA or EOB (Table 6); however, there may have been an effect for several measures with p<0.10 (effect sizes of -1.5 to -2.2). Results of regression models controlling for age as a covariate reveal similar patterns of significance and directionality of results for the TR group. In the TH group, crude regression results show statistically significant differences for AM-ULA, and the two UNB tests with results favoring IMU controls at End of A, however these differences were no longer significant after controlling for age (S1 Table). Residual plots (S1 and S2 Figs) showed that while the residuals demonstrated fairly good patterns of normality and homoscedasticity, regression models of QuickDASH, and the TH subgroups may not have met the normality assumptions.

## Discussion

This study quantified outcomes of EMG-PR control and IMU control of the DEKA Arm and compared outcomes of the DEKA Arm to conventional prostheses for subjects who used a personal prosthesis. It also compared outcomes by amputation level for groups of subjects who utilized EMG-PR control and IMU control of the DEKA Arm.

Our analyses showed that subjects using EMG-PR had mixed results in terms of perceived activity difficulty, more use of the prosthesis for everyday tasks, but worse dexterity, lower prosthetic satisfaction, slower activity performance at EOA as compared to Baseline testing. The only difference between EMG-PR and Baseline outcomes at EOB was less perceived difficulty in activity performance as measured by the PSFS after EMG-PR use.

In contrast, subjects using the IMU controls had no differences in dexterity, lower self-reported difficulty in activity, but demonstrated slower activity performance at EOA as compared to Baseline. At EOB, subjects using IMUs had improved activity performance, improved self-reported disability scores, lower self-reported difficulty in activity performance (as measured by the PSFS) and more use of the prosthesis during everyday activities, but demonstrated slower activity performance and more pain as compared to Baseline. Of note, at the end of home use, at their final testing visit, EMG-PR users reported that they were not engaging the DEKA Arm for any of the activities listed in the UEFS measure. This was a decline in prosthesis engagement as compared to their baseline function. In contrast, users of the IMU controlled device reported using the prosthesis in more UEFS activities than they did at baseline. Subjects in our study used EMG-PR or IMU controls to operate up to 4 functions of the RC DEKA Arm and up to 6 functions for users of the HC DEKA Arm. Functions controlled included powered DOF of the prosthesis as well as toggle grip selection that allowed users to choose between the DEKA Arm’s 6 pre-programmed grasps. Comparisons of EMG-PR to IMU of the RC DEKA Arm suggested that subjects achieved better outcomes using IMUs as compared to EMG-PR, and received fewer hours of prosthetic training. IMU users demonstrated better dexterity, as measured by 3 JTHFT subtests at EOA (large ES), and 4 subtests at EOB (large ES). They also had improved activity performance at EOA (large ES), lower ratings of disability at EOA (moderate ES) and EOB (large ES), and improved self-reported difficulty of activity (UEFS) at EOB as well as greater use of the prosthesis during activities (large ES). Additionally, ratings of prosthesis satisfaction were significantly better for the IMU group as compared to EMG-PR group at both time periods (large ES).

No differences between groups were observed for users of the HC DEKA Arm. While we did not find any statistically significant differences between groups of HC users, the raw data shows a trend towards improved dexterity for the EMG-PR users with better scores on several JTHFT tests, lower self-reported disability and activity difficulty (see Table 6). Given our small sample sizes, our study should be viewed as exploratory and hypothesis generating. Larger studies, which are adequately powered, are needed to determine whether EMG-PR controls offer significant benefits as compared to IMU control of the DEKA Arm.

### Limitations

The results of this study should be interpreted with caution due to limitations in the study design and sampling strategy. The EMG-PR to DEKA Arm interface was developed specifically for this study, and utilized two prototypes. Our team experienced technical issues originating in the controls interface which may have influenced the results. A separate paper explores the user perspective on using the EMG-PR controlled DEKA Arm as well as the impact of repeated technical problems requiring repair and adjustment. [24] It is possible, that future iterations of an EMG-PR controls interface for the DEKA Arm may minimize some of these issues and may lead to improved outcomes. Future research is needed to confirm or refute our findings, should a newer prototype become available.

Findings regarding the comparability of EMG-PR and IMU for the users of the HC Arm relied on a sample of only 9 subjects, only 2 whom utilized EMG-PR. These two subjects had undergone Targeted Muscle Reinnervation (TMR), [25, 26] and therefore, their results cannot be generalized to other persons with TH amputation who have not had TMR. Further studies are needed to evaluate the comparative effectiveness of EMG-PR control of the HC DEKA Arm for persons who have not had TMR. The conclusions that can be drawn from our data are also limited by the study design. The control systems for participants within the IMU and EMG-PR groups were not homogenous, and we did not perform any sub-analyses to determine if there were differences within groups, or across sub-groups. Some participants in the IMU group used IMU controls alone, while others used IMUs in combination with supplemental controls such as pneumatic bladders, linear transducers, and sEMG. Participants in the EMG-PR group used two different prototypes of PR control: with and without EMG-PR control of grip selection. Future studies could explore outcomes across these smaller subgroups.

This was a quasi-experimental study where subjects were not randomly assigned to control methods. We compared outcomes for each control type to outcomes achieved with subjects’ personal prosthesis. While we believe that these comparisons are informative, they have limitations due to the variety of devices used in our study, and the non-equivalent exposure and training with the personal prosthesis. Subjects had relatively short exposure to the DEKA Arm through the study, and in some cases had many years of experience with their own prostheses–and so were likely to be fully acclimated to their own devices. To help mitigate this, subjects had structured occupational therapy training during the study, where they were trained to use the DEKA prosthesis and controls. Some of our subjects may not have had similar prosthetic training with their own prosthesis, and what training they had received occurred well before the study. Thus, we cannot say with certainty that, given similar amounts of training and experience, the outcomes comparing the personal prosthesis to the DEKA Arm (with either control method) would have been the same. Studies with stronger designs that might account for factors including years of prosthesis experience and amount of prosthetic training are, in our view, not feasible, given the small numbers of upper limb amputees and the costs of conducting multi-site, randomized controlled trials.

Additionally, the comparison between EMG-PR and IMU groups use a non-equivalent comparison groups. We attempted to control for this non-equivalence by stratifying our analyses by DEKA Arm configuration level, and by modeling the outcomes after controlling for observed group differences by age. Although we found that our results comparing outcomes by control group were unchanged, even after controlling for age, we recognize that there may have been unobserved differences by group that affected outcomes achieved during the study.

## Conclusion

Scores of measures of dexterity, prosthetic satisfaction, speed of activity performance when using an EMG-PR controlled DEKA Arm after in-laboratory training were worse than Baseline scores measured using a conventional prostheses. After home use experience, EMG-PR users reported less difficulty in activities. In comparison, users of an IMU controlled DEKA Arm had equivalent outcomes as compared to baseline except for speed of activity performance which was slower. After home use, users of the IMU controlled DEKA Arm had better activity performance, less disability and activity difficulty. However, speed of performance was still slower than measured with conventional prostheses at Baseline. Direct comparisons between groups using EMG-PR and IMU control of the DEKA Arm showed that for subjects with TR amputation, EMG-PR was associated with worse dexterity and activity performance. There were no differences observed by control type for subjects with TH amputation.

These findings suggest that for persons with TR amputation, IMUs are a more effective control method for the DEKA Arm as compared to the EMG-PR prototypes employed in this study. In its current state, the clinical use of EMG-PR to control the DEKA Arm is premature. Further research is needed to confirm the findings generated in this study. Future study should include a larger sample of TH amputees and with new EMG-PR prototypes when they become available.

## Supporting information

S1 Fig Residual vs Predicted and Q-Q plots for regressions controlling for age: TR Subgroup. (EPS)

S2 Fig Residual vs Predicted and Q-Q plots for regressions controlling for age: TH Subgroup. (EPS)

S1 Table (DOCX)

S2 Table (DOCX)

S1 File IRB initially approved protocol. (PDF)

S2 File IRB approved modified protocol. (PDF)

S3 File Clinical trials protocol updated. (PDF)

S4 File TREND checklist. (PDF)
