# Supplementary material for: EMG pattern recognition compared to foot control of the DEKA Arm
Source: PLoS One. 2018 Oct 18;13(10):e0204854. doi: 10.1371/journal.pone.0204854 (PMC6193636; doi:10.1371/journal.pone.0204854)
Supplement: S1 Table — (DOCX) [file pone.0204854.s003.docx]

**APPENDIX A.**

|  | **End of A (N=27)** | | | | |  |  |
| --- | --- | --- | --- | --- | --- | --- | --- |
|  | **EMG-PR (N=9)** | | **Other (N=17)** | | **Wilcoxon Ranksum** | **t-test** | **Regression controlling for age** |
|  | **N** | **Mn (sd)** | **N** | **Mn (sd)** | **P** | **P** | **P** |
| **Dexterity** |  |  |  |  |  |  |  |
| Jebsen-Taylor Hand Function (JTHFT) items/sec |  |  |  |  |  |  |  |
| JTHFT: Writing | 9 | 0.41 (0.13) | 17 | 0.44 (0.18) | 0.63 | 0.5894 | 0.4405 |
| JTHFT: Page Turning | 9 | 0.06 (0.03) | 17 | 0.07 (0.03) | 0.22 | 0.4060 | 0.6848 |
| JTHFT: Small items | 9 | 0.04 (0.04) | 17 | 0.10 (0.09) | 0.07 | 0.0581 | 0.0994 |
| JTHFT: Feeding / Eating | 9 | 0.05 (0.05) | 17 | 0.09 (0.06) | 0.06 | 0.0779 | 0.1071 |
| JTHFT: Checkers | 9 | 0.05 (0.05) | 17 | 0.11 (0.06) | **0.02** | **0.0188** | **0.0441** |
| JTHFT: Light Cans | 9 | 0.10 (0.08) | 17 | 0.31 (0.11) | **<0.001** | **<0.0001** | **0.0002** |
| JTHFT: Heavy Cans | 9 | 0.13 (0.10) | 17 | 0.33 (0.11) | **<0.001** | **0.0003** | **0.0007** |
| **Activity** |  |  |  |  |  |  |  |
| AM-ULA | 9 | 1.6 (0.3) | 17 | 2.0 (0.4) | **0.01** | **0.0179** | **0.0394** |
| UNB: Spontaneity | 9 | 3.0 (0.5) | 16 | 3.2 (0.5) | 0.41 | 0.4063 | 0.4173 |
| UNB: Skill | 9 | 2.8 (0.6) | 16 | 3.0 (0.6) | 0.46 | 0.4513 | 0.4765 |
| T-MAP | 7 | 494.4 (264.1) | 14 | 598.2 (475.2) | 0.80 | 0.5998 | 0.1907 |
| BAM-ULA summary (new) | 8 | 6.9 (2.2) | 12 | 8.3 (1.4) | 0.14 | 0.1081 | 0.1921 |
| **Self-reported function** |  |  |  |  |  |  |  |
| QuickDASH | 9 | 27.3 (8.3) | 17 | 21.3 (14.4) | **0.05** | 0.2671 | 0.2159 |
| Upper Extremity Functional Scale (UEFS) | 7 | 46.2 (9.2) | 14 | 44.2 (7.3) | 0.48 | 0.6081 | 0.5183 |
| UEFS use | 9 | 0.5 (0.3) | 17 | 0.6 (0.4) | 0.50 | 0.5891 | 0.4008 |
| Patient Specific Functional Scale (PSFS) | 9 | 5.8 (2.0) | 17 | 5.4 (1.9) | 0.78 | 0.6977 | 0.6058 |
| **Quality of life etc.** |  |  |  |  |  |  |  |
| Wong-Baker Pain Scale | 9 | 0.8 (0.7) | 17 | 0.8 (1.2) | 0.62 | 0.9160 | 0.8445 |
| Quality of Life (QOL) Scale | 9 | 5.5 (0.5) | 17 | 5.8 (0.8) | 0.42 | 0.3440 | 0.2103 |
| Community integration CRIS-CAT |  |  |  |  |  |  |  |
| Extent of Limitations | 9 | 53.9 (5.6) | 17 | 55.7 (10.6) | 0.61 | 0.6493 | 0.6527 |
| Perceived Limitations | 9 | 49.6 (5.3) | 17 | 54.8 (15.4) | 0.62 | 0.3396 | 0.3822 |
| Satisfaction with Participation | 9 | 48.3 (3.9) | 17 | 51.4 (6.6) | 0.27 | 0.2142 | 0.2371 |
| TAPES Satisfaction Scale | 9 | 2.9 (0.6) | 17 | 3.5 (0.7) | **0.03** | **0.0352** | **0.0194** |

**S1 Table. Comparison of outcomes by control type at End of A with and without controlling for age: transradial amputees**

**Long Caption:** S1 Table shows the p-values for Wilcoxon Ranksum tests, t-tests and regressions on the effect of control type after controlling for age for participants with transradial amputation. P-values derived from the regressions without controlling for age are identical to those achieved through t-tests.
