# Supplementary material for: EMG pattern recognition compared to foot control of the DEKA Arm
Source: PLoS One. 2018 Oct 18;13(10):e0204854. doi: 10.1371/journal.pone.0204854 (PMC6193636; doi:10.1371/journal.pone.0204854)
Supplement: S2 Table — (DOCX) [file pone.0204854.s004.docx]

|  | **End of A (N=10)** | | | | |  |  |
| --- | --- | --- | --- | --- | --- | --- | --- |
|  | **EMG-PR (N=2)** | | **Other (N=8)** | | **W R-S** | **t-test** | **Regression controlling for age** |
|  | **N** | **Mn (sd)** | **N** | **Mn (sd)** | **P** | **P** | **P** |
| **Dexterity** |  |  |  |  |  |  |  |
| Jebsen-Taylor Hand Function (JTHFT) items/sec |  |  |  |  |  |  |  |
| JTHFT: Writing | 2 | 0.33 (0.10) | 8 | 0.27 (0.11) | 0.40 | 0.4544 | 0.7077 |
| JTHFT: Page Turning | 2 | 0.07 (0.02) | 8 | 0.04 (0.02) | 0.09 | 0.0607 | 0.0618 |
| JTHFT: Small items | 2 | 0.04 (0.02) | 8 | 0.05 (0.02) | 0.40 | 0.2940 | 0.1533 |
| JTHFT: Feeding / Eating | 2 | 0.09 (0.04) | 8 | 0.07 (0.05) | 1.00 | 0.7122 | 0.4842 |
| JTHFT: Checkers | 2 | 0.04 (0.02) | 8 | 0.04 (0.04) | 0.89 | 0.9751 | 0.9496 |
| JTHFT: Light Cans | 2 | 0.08 (0.02) | 8 | 0.08 (0.06) | 0.53 | 0.9809 | 0.7489 |
| JTHFT: Heavy Cans | 2 | 0.13 (0.07) | 8 | 0.09 (0.05) | 0.53 | 0.3438 | 0.8853 |
| **Activity** |  |  |  |  |  |  |  |
| AM-ULA | 2 | 19.2 (3.5) | 8 | 14.5 (1.8) | 0.07 | **0.0224** | 0.0881 |
| UNB: Spontaneity | 2 | 3.5 (0.1) | 8 | 2.9 (0.3) | 0.07 | **0.0426** | 0.2704 |
| UNB: Skill | 2 | 3.3 (0.1) | 8 | 2.7 (0.3) | 0.07 | **0.0237** | 0.2870 |
| T-MAP | 2 | 740.0 (134.4) | 8 | 756.6 (220.1) | 0.89 | 0.8819 | 0.4614 |
| BAM-ULA summary (new) | 2 | 8.5 (0.7) | 6 | 6.5 (1.1) | 0.14 | 0.0498 | 0.27812 |
| **Self-reported function** |  |  |  |  |  |  |  |
| QuickDASH | 2 | 28.4 (4.8) | 8 | 28.7 (6.7) | 0.98 | 0.9569 | 0.6268 |
| Upper Extremity Functional Scale (UEFS) | 2 | 44.5 (5.3) | 8 | 43.7 (4.4) | 1.00 | 0.8291 | 0.8656 |
| UEFS use | 2 | 0.7 (0.5) | 8 | 0.7 (0.3) | 1.00 | 0.8518 | 0.9635 |
| Patient Specific Functional Scale (PSFS) | 2 | 7.2 (0.5) | 8 | 5.3 (2.1) | 0.27 | 0.2719 | 0.1897 |
| **Quality of life etc.** |  |  |  |  |  |  |  |
| Wong-Baker Pain Scale | 2 | 1.0 (1.4) | 8 | 1.3 (0.7) | 1.00 | 0.7128 | 0.7482 |
| Quality of Life (QOL) Scale | 2 | 5.1 (1.3) | 8 | 6.2 (0.6) | 0.38 | 0.0846 | 0.3792 |
| Community integration CRIS-CAT |  |  |  |  |  |  |  |
| Extent of Limitations | 2 | 45.5 (17.7) | 8 | 56.0 (6.1) | 0.51 | 0.1550 | 0.3687 |
| Perceived Limitations | 2 | 49.5 (10.6) | 8 | 54.9 (9.1) | 0.42 | 0.4848 | 0.364 |
| Satisfaction with Participation | 2 | 48.0 (9.9) | 8 | 56.4 (9.4) | 0.44 | 0.2946 | 0.396 |
| TAPES Satisfaction Scale | 2 | 3.5 (0.6) | 8 | 3.9 (0.9) | 0.49 | 0.6184 | 0.6685 |

**S2 Table Comparison of outcomes by control type at End of A with and without controlling for age: transhumeral amputees**

**Long Caption:** S2 Table shows the p-values for Wilcoxon Ranksum tests, t-tests and regressions on the effect of control type after controlling for age for participants with transhumeral amputation. P-values derived from the regressions without controlling for age are identical to those achieved through t-tests.
