# Supplementary material for: EMG pattern recognition compared to foot control of the DEKA Arm
Source: PLoS One. 2018 Oct 18;13(10):e0204854. doi: 10.1371/journal.pone.0204854 (PMC6193636; doi:10.1371/journal.pone.0204854)
Supplement: S2 File — (PDF) [file pone.0204854.s006.pdf]

Background and Significance

The number of patients with upper extremity amputation who are cared for in Veterans Administration facilities is increasing as a result of injuries sustained in recent conflicts. The majority of these amputees will separate from active duty and enroll in VA healthcare. Returning these amputees to full participation in work, leisure and social activities will be challenging given the limitations of commercially available upper limb prosthetics. Currently, the individuals with upper limb amputations must perform complex motions with control that is slow, cumbersome and unnatural. Furthermore, amputees have no direct sensory feedback with their prostheses and only receive feedback on the limb performance by watching or in some cases listening to the sounds generated by motors.

Given these challenges, representatives of the VA and DARPA have called for more collaboration between the two entities in meeting the needs of individuals who have lost their arms and highlighted the need for additional research on upper extremity prosthetics. DARPA funded the development of the DEKA Prosthetic Arm which incorporates major technological advances such as flexible socket design, and innovative control features and software that together enable enhanced functionality that promises to surpass any currently available prosthetic device.

The DEKA Gen 3 Arm

There are three versions of the DEKA Arm: shoulder configuration (SC, also known as shoulder disarticulation (SD)); humeral configuration (HC also known as transhumeral (TH)) and radial configuration (RC also known as transradial (TR)). Photographs and characteristics of the Gen 2 version are shown in the Figure 1. All versions have the following common features: multiple configurable hand grips, simultaneous joint control, intuitive control schemes, control inputs flexibility, compliance and afferent feedback. DEKA has conducted engineering tests to characterize/verify performance and refined “strap-and-go” arm control methods. The DEKA design incorporates improved end point control, and has added and refined multiple alternative control methods. They have built and tested smaller haptic systems and built smaller Gen 2 tactors with separate, mountable electronics. They also have implemented a simple vibration tactor.

	**Shoulder Configuration**	**Humeral Configuration**	**Radial Configuration**
	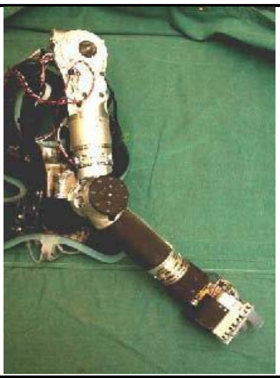	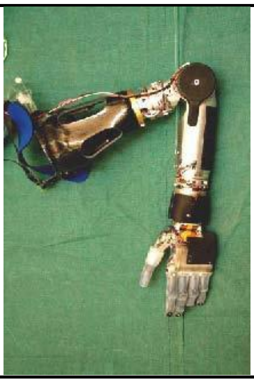	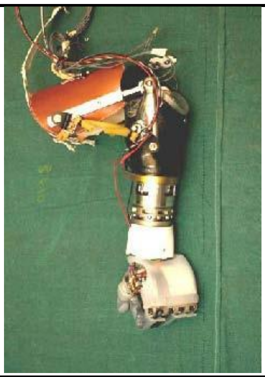
**Degrees of Freedom<br>Total<br>Powered**	**<br>15 (6 arm, 9 hand)<br>10 (6 arm, 4 hand)**	**<br>13 (4 arm, 9 hand)<br>8 (4 arm, 4 hand)**	**<br>11 (2 arm, 9 hand)<br>6 (2 arm, 4 hand)**
**Weight**	**8.0 lbs**	**5.6 lbs**	**2.5 lbs**
**Control**	**Foot pads, Shoulder joystick, Shoulder bladders, EMG switch**	**Foot pads, EMG switch**	**Foot pads, EMG Pattern recognition**

Figure 1. Versions and Characteristics of the Gen 2 DEKA Arm

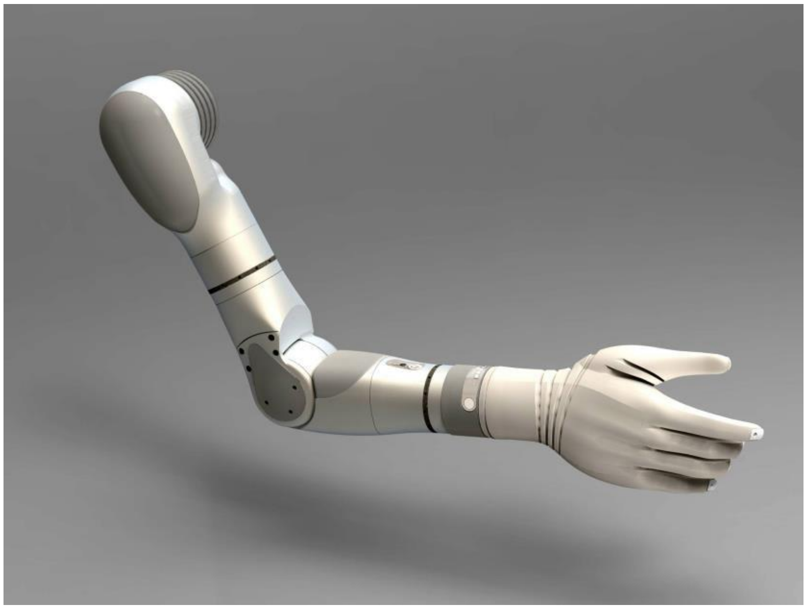

Figure 2. Schematic View of Full (Shoulder Configuration) Gen 3 Arm

The Gen 3 Arm system (Figure 2) incorporates several new features and changes such as: a compound wrist that includes radial/ulnar deviation with extension/flexion, improved external battery and charger, improved tactor, changes in wireless communication protocol, improved grip strength and hand speed, changes in finger grip trajectory, hardware changes to wrist and shoulder joint design, changes to connectors and cables, refinement of EMG and IMU sensors, changes to the user notification system, and improvements to the prosthetist user interface. These updates do not change the FDA risk determination (i.e. it is considered to be the same prosthetic Arm system). The DEKA Gen 3 Arm began clinical studies in late Spring 2011.

Significance of the study
The proposed study builds on the VA's ground-breaking example of a successful DARPA-VA research collaboration that will continue the VA's tradition of funding innovations and advances in prosthetic technology. This VA led home study will demonstrate the VA's commitment to advance cutting-edge prosthetic technology. Information gleaned from this study will be critically important in moving the DEKA Arm closer to commercialization and successful dissemination and deployment within the VA system of healthcare. Analyses conducted in the proposed study will be used to identify amputees who are appropriate candidates for advanced prosthetic devices as well as those who are likely to be poor candidates. The study will assess usefulness and acceptability in a home environment. Data from this study will help inform future VA Prosthetics and Sensory Aids clinical guidelines for prescription of advanced upper limb prosthetic technology.

Specific Aims
The DEKA Arm, developed through DARPA's "Revolutionizing Prosthetics" Program, incorporates major technological advances such as active volume controlled socket design, and innovative control features and software that together enable enhanced functionality that promises to surpass any currently available prosthetic device. A Memorandum of Agreement between the VA and DARPA paved the way for a two year VA clinical trial to optimize the Gen 2 prototype DEKA Arm System. This study provided data to inform DEKA's development of the Gen 3 prototype. Evaluation of the Gen 3 prototype is underway,. Although DEKA has conducted a limited number of take home studies of the Gen 2 device, the VA studies of the DEKA Arm to date have only been conducted in-lab, meaning that VA subjects have used the prosthesis only under supervised conditions and were not allowed to take the device home and integrate it into their daily use. Thus, key outcomes including the extent of use or adoption of the arm for activities at home and the impact of the advanced prosthetic device on subjects' quality of life have not been previously examined. The next step in determining the usefulness and acceptability of this advanced prosthetic device is to conduct studies of its home usage. The overall objective of the proposed study is to examine the feasibility, acceptance and benefits

of home use of an advanced upper limb prosthetic device as well as the logistical support requirements utilized during 3 months of home usage. The specific objectives of this quasi-experimental, time series study are to:

1. Identify and describe upper limb amputees who would be appropriate candidates for home use of this advanced prosthesis, as well as those who would not be appropriate.
All participating subjects will enroll in Part A of the study, which will involve supervised, in-laboratory training, as well as supervised community based outings. Based upon the findings from Part A, study staff will classify participants as appropriate or not appropriate for participation in Part B (the home usage portion of the study). This classification will be based upon a specified set of criteria established to determine potential for safe home usage, as well as the completion of all aspects of Part A study activities. Safe home use criteria will include elements such as skillfulness of prosthetic use, independence in daily device maintenance and storage, and safety awareness. Final determination of appropriateness for home use will be made after a home visit is conducted to assess the appropriateness of the home environment. We will attempt to identify any factors (such as results of neuropsychological testing) that can be used to predict those subjects who are deemed as not appropriate candidates for home use.

2. Compare the extent of use of the existing prosthesis to that of the DEKA Arm and quantify the impact of home use of the DEKA Arm on device satisfaction, performance of functional activities and the user's quality of life.
Subjects will be asked to maintain daily logs detailing usage of their current prostheses. Additionally, data logs will be generated in the DEKA Arm software. This data will be collected several weeks prior to in-lab training with the DEKA Arm. Measures of existing prosthetic activity will be compared with those of home use of the DEKA Arm to determine the extent of adoption.

3. Compare the outcomes of users of pattern recognition to the outcomes of users without.
We will use a time series design to examine changes in device satisfaction, performance of functional activities and quality of life throughout the study.

4. ***Quantify the amount and type of technical support and repairs needed during the study, and estimate the number of home study days lost due to service/repair.***
We will gather data on technical support usage, including number and content of calls and email exchanges with DEKA support and number and type of repairs needed during the study. This information will be useful in estimating service needs for clinical deployment.

This study will provide important information to help determine which amputees are appropriate candidates for this advanced prosthetic device, as well as provide data on usefulness and acceptability in a home environment. Data from this study will help inform the development of clinical guidelines for prescription of advanced upper limb prosthetic technology. Currently, VA Prosthetics and Sensory Aids has few, if any, clinical guidelines to inform prescription of advanced technologies, determine appropriate candidates or readiness for home use of prosthetic devices.

Need for future research

The VA Study to Optimize the DEKA Arm was conducted entirely in-laboratory, meaning that all usage of the DEKA Arm was supervised by study staff and subjects did not take the arm home, nor were any subjects left

alone while wearing the arm. Although our training protocol involves performance of many functional activities such as cooking, simulated housekeeping and eating, subjects did not have the opportunity to utilize the arm in their own environment, or without supervision. Thus, the study was unable to gather data on the impact of DEKA Arm usage on quality of life. Nor was this study able to glean information on adoption of the arm in home use and/or compare data on wear time of the DEKA Arm in the home environment. The proposed study will provide the opportunity to examine these critical areas of prosthetic use.

Research Design and Methods

Design

This proposed study will employ a quasi-experimental, time series design to provide the data needed to compare usage of existing prosthesis to the of the DEKA Arm, and assess the acceptance and benefits of home usage. This study will involve two parts. Part A will involve in-laboratory training and supervised community based outings using the DEKA Arm. A selection of Part A participants will be invited to participate in Part B testing which will involve a 3 month trial of home use with biweekly study visits.

Acquisition of DEKA Arms

Gen 2 and Gen 3 Arms for the VA Study to Optimize the DEKA Arm were provided under the terms of CRADAs between each VA Medical Center and DEKA. These activities were coverable under the terms of the DEKA-DARPA contract to develop the advanced prosthetic arms. Because the DARPA contract is ending, a new mechanism for obtaining the DEKA Arms will be utilized. The PVAMC will contract with DEKA directly to purchase arms for this study. Negotiations with DEKA are underway and this multi-year contract is expected to be executed by 10/1/11.

Sample

Part A: We expect that we may need to enroll up to 100 amputees across all sites in order to yield complete Part A data on 40 subjects. This is due to anticipated screen failures (estimated at 20%) and the likelihood that some 20% of subjects (based upon prior study experience) who do meet eligibility criteria will not be able to participate in all research activities. Additionally, we will purposefully sample subjects to insure that we have an even distribution of subjects at each level of DEKA Arm fit during Part B (10 SD, 10 TH, 10 TR).

Note: Subjects who have completed study participation may be invited, at the request of the Principal Investigator, to participate a second time in order to be set up with and evaluate an alternative controls scheme than what they had used previously. These subjects will need to be consented again and complete all study activities including screening.

Part B: We expect that up to 20% of Part A participants may not be considered appropriate for home study usage. Thus, we expect that up to 36 participants who have completed Part A of the study will be invited to participate in the home study portion (Part B) of the study. We will purposefully select subjects who have completed Part A and who are judged to be appropriate candidates for home use (see Part A Preliminary Determination of Appropriateness for Unsupervised, Home Device Use). Our goal is to have complete data on up to 30 subjects with the Gen 3 DEKA Arm. We plan to include approximately10 subjects fit at each of the 3 levels of amputation (SD, TH, TR). We estimate that 20% (or 4 subjects) may terminate the study early.

For both parts of the study, an estimated 80% of subjects will be veterans enrolled in the VHA or DOD healthcare systems. Approximately 20% of subjects may be non-veterans whose participation may be needed to meet sampling goals for amputees with higher levels of limb loss, and to insure the inclusion of female amputees. Subjects who have participated in the VA Study to Optimize the DEKA Arm or the VANYHHS pilot study to optimize the DEKA Arm will be eligible to participate in this study.

<u>Inclusion Criteria (Parts A and B):</u>

- All subjects must be at least 18 years old and have single or bilateral upper limb amputation.
- Subjects must be able to understand the requirements of the study and sign an Informed Consent Form

and HIPAA Authorization Form.
- Subjects will include those who are current users of any type of prosthetic device (body powered, electric or hybrid), non-users of devices who have been previously fit with a device, but have chosen not to wear it, as well as new users of devices.
- To participate in the study all subjects must have active control over one or both ankles, OR have an appropriate number of myoelectric and/or other control sites (as determined by the Principal Investigator in conjunction with the research team) to allow adequate prosthetic controls configuration.

Inclusion Criteria Part B only:

- Completion of all Part A study activities.
- Meets all criteria for Preliminary Determination of Appropriateness for Unsupervised, Home Device Use (see Preliminary Determination of Appropriateness for Unsupervised, Home Device Use).

Exclusion Criteria (Parts A and B):

- Amputees with elbow disarticulation, wrist disarticulation and partial hand amputations will be excluded.*
- Amputees will be excluded if the length of their residual limb would prohibit socket fitting, as determined by the study prosthetist.
- Persons with significant uncorrectable visual deficits that would impair the ability to see the prosthesis and those who have major communication or neurocognitive deficits will be excluded.
- Persons with skin conditions such as burns or poor skin coverage as well as those with severe contractures that prevented prior prosthetic wear will be excluded.
- Persons with electrically controlled medical devices including pacemakers, and implanted defibrillators will be excluded.
- Persons with neuropathy, uncontrolled diabetes, who are receiving dialysis, have insensate feet, severe phantom pain or a history of skin ulcers or any other significant comorbidity which would interfere with the study will be excluded.
- Those with severe circulatory problems including peripheral vascular disease and pitting edema will be excluded.
- Persons with cognitive deficits or mental health problems that would limit their ability to participate fully in the study protocol will be excluded.
- Women who are pregnant or who plan to become pregnant in the near future will also be excluded.
- We will exclude those amputees who work for prosthetic companies that may be considered competitors for the DEKA Arm technology in the future.
- Persons taking medication which poses a risk for operation of heavy equipment will be excluded.

After 7/1/16, all amputees with shoulder disarticulation or forequarter amputation will be excluded.

Impaired Decision-making Capacity:

In cases where decision-making capacity of a potential research subject is questioned, the modified Dysken tool will be used as an assessment tool to evaluate capacity to provide informed consent. Subjects must answer at least 70% of questions on the screening tool correctly in order to be considered as having sufficient understanding of the study to provide informed consent.

Sampling strategy

Subjects will be purposefully sampled to include participants at each of three levels: transradial, transhumeral, shoulder disarticulation/forequarter level. Our goal is to have a heterogeneous sample with a mix of unilateral and bilateral amputees, regular prosthetic users as well as non-users. We hope to include female amputees at each level, if at all possible. We believe that this projected sample size will be adequate to achieve our goals. Previous studies show that 80% of major usability problems (as judged by need for technical support and repair) can be detected within the first 5 cases.[10] The FDA suggests that data from about 10 individuals from a homogenous population is typically sufficient to discover most types of usability concerns.[11] However, with greater heterogeneity, a greater sample size may be desirable.[11] Thus, experts in usability testing recommend

the number of subjects should not be fixed apriori, but rather continue, if possible, until no issues are uncovered. [10] Given this, our goal is to sample between 5 and 10 subjects from each amputation level.

Subject Recruitment

A number of methods will be utilized to recruit subjects into the study. First, clinical staff from study sites will inform potentially eligible subjects about the study and invite them to participate. Second, email notifications regarding the study will be sent periodically to national list-servs for prosthetic and rehabilitation staff. Third, approved flyers and brochures will be distributed at VA/DoD medical centers, private prosthetic treatment centers, and other locations that upper limb amputees might frequent. Fourth, we will distribute press-releases to news groups as well as consumer organizations including the Amputee Coalition of America, Wounded Warriors, and the Disabled Veterans of America. Thus, potential subjects who show an interest in participating can learn more about the study from this informational material. Finally, subjects who participated in earlier VA studies of the DEKA Arm, who consented to be contacted in the future may be informed about the new study and invited to participate.

Research Sites

The proposed study will be coordinated by Dr. Linda Resnik at the Providence VA Medical Center (PVAMC). Dr. Resnik is the Principal Investigator for the multisite VA Study to Optimize the DEKA Arm. As such, her team has acquired the expertise and experience to lead the proposed study. Data collection will take place at 3 sites, including 2 VA Medical Centers and 1 DoD site. Given that upper limb amputees comprise less than 3% of the amputee population and few VA/DoD prosthetists have extensive experience in this area, we will utilize sites who participated in the VA Study to Optimize the DEKA Arm. If additional funding becomes available, there is the possibility that we may include one or two additional research sites to achieve our subject recruitment goals. New sites will be required to have certified prosthetists who are very experienced with upper extremity prosthetic fitting and fabrication, fabrication facilities, and the availability of an experienced therapist to lead the training. We expect to begin Year 1 of the study with the following 3 experienced sites: VANYHHS (Manhattan), James Haley VA (Tampa) and Center for the Intrepid (CFI) at Brooke Army Medical Center (San Antonio.). After 7/1/2016, the James Haley VA (Tampa) will be the only site actively recruiting participants.

Training and Supervision of Study Staff at each Site

Proper fit of the prosthetic socket requires a great deal of advanced clinical decision-making and prosthetic fabrication skill. Proper socket fit is an absolute requirement for successful set-up and use of the DEKA Arm system. Because the fitting and fabrication of the DEKA Arm’s dynamic socket may be new to study prosthetists, we plan to provide training, supervision and support for prosthetists to increase the chances of a successful result. Before a new site begins data collection, the site prosthetist will participate in hands-on training to observe fit and fabrication of the dynamic socket and set-up of a DEKA Arm system. Following that observation, each prosthetist will fit the first subject of each level of amputation under the supervision of a more experienced study prosthetist. Additional subjects (beyond the initial subject at each amputation level) will be fit under supervision as needed, based on the complexity of the case and prosthetist judgment. Throughout the remaining study data collection period, the site prosthetist will consult with the study’s lead prosthetist and/or prosthetic consultant/s and with DEKA engineers by telephone, video consultation and in-person as needed for troubleshooting and technical advice.

Prior to initiation of study activities at each site, the study therapist (who will be either a PT or an OT), will be trained in the study training and testing procedures by Dr. Resnik in conjunction with experienced therapists (who participated in VA Study to Optimize the DEKA Arm). Dr. Resnik and/or the study Project Manager will oversee implementation of training and testing protocols. Video and teleconferences between the study personnel will be held at regularly scheduled intervals (e.g. monthly) and as needed for monitoring of fidelity to the study protocol and troubleshooting.

Overview of Study Procedures

Part A – Current Device Usage Monitoring and Supervised Training

Each subject's involvement in Part A of the study will require 2 weeks of home device use monitoring with their existing prosthesis and between 18-34 supervised visits at the site. The majority of visits will be for training with the DEKA Arm. In addition, some subjects may require an additional 3-5 visits beyond the estimated 18-34 visits because of a need to modify their prosthetic socket or reconfigure their controls.

Part A study procedures may be broken down into two components: 1) baseline testing and current device usage monitoring and 2) fitting/set-up and 3) training, in order to allow subjects to fit the study activities into their schedule. The estimated timeline of participation for Part A study activities is shown in the Gannt chart below.

**Estimated Timeline of Study Activities: Part A (Weeks)**												
**Activities**	1	2	3	4	5	6	7	8	9	10	11	12
Consent Process												
Medical Screening												
Baseline Data Collection												
Current device usage monitoring												
Pre-Prosthetic Training												
Prosthetic fit and set up												
Training sessions (average 3x/week)												
Preliminary Determination of Appropriateness for Home Use												

<u>Part B - Take Home Portion of the Study</u>

Subjects who complete all Part A study activities and who are determined to be appropriate candidates for unsupervised home device use will be invited to participate in the Take Home Portion of the study (Part B). Prior to initiation of any take home activities, the study staff will conduct an assessment of the subject's home environment to make a final determination of appropriateness of unsupervised, take home usage. Once cleared for home use, Part B, subjects will return to the sites on a biweekly basis for study visits and will be asked to maintain daily usage logs between visits. At the conclusion of Part B subjects will have a final session of data collection. It is estimated that Part B participation will last a minimum of 13 weeks. This timeline, shown in the Gannt chart below may be extended in the event that the arm is broken and requires repair. If this should occur, and the subject agrees, the length of the home study time will be extended to adjust for the number of days lost.

**Estimated Timeline of Study Activities: Part B (Weeks)**													
**Activities**	1	2	3	4	5	6	7	8	9	10	11	12	13
Consent for Part B													
Home Visit/Final Determination of Appropriateness													
Begin take home activities													
Daily Usage Logs													

Biweekly study visits													
Final data collection session													

Part A Procedures

Part A - Visit 1

Consent process

After initial telephone screening to assess eligibility criteria, each potential subject will be scheduled for an in- person assessment which will be held in a private area. Before the assessment begins, the site PI, research assistant, or prosthetist will explain the study protocol. Subjects will be asked to make a commitment to be available for all study activities. Individuals will be given adequate time to review and comprehend all information about the study before agreeing to take part, minimizing the possibility of coercion and undue influence.

Please note: (1) subjects who enrolled in the Gen 2 phase of the study and are returning for the Gen 3 phase of testing will be given a new subject number and will complete all study visits. (2) Subjects who enrolled in the VA Study to Optimize the DEKA Arm and / or the Home Study of An Advanced Upper Limb Prosthesis, who are returning to participate, be set up, and evaluated with an alternative controls scheme will be given a new subject number.

Medical screening

The site physician will conduct an in-person medical screening to confirm that inclusion/exclusion criteria are met. If the subject is found to meet the inclusion/exclusion criteria, is willing to participate,provides written consent, and is considered a viable candidate for pattern recognition (Coapt) use -if enrolling in to be evaluated with this alternative controls scheme- they will then be enrolled in the study. After enrollment, the study staff will provide detailed teaching and orientation material about the DEKA Arm system. Subjects will be scheduled for prosthetic fitting and set- up as scheduling and Arm availability permits. The remainder of activities for Visit 1 may be completed in the same session, or if scheduling requires, may be completed in a separate session.

Collection of baseline data

After enrollment, the prosthetist and therapist will collect baseline data on current prosthetic use (if any), prosthetic componentry, history of falls, and study measures. Diagnostic information regarding traumatic brain injury (TBI) and/or history of head injury will be recorded. Subjects will complete a brief neuropsychological screening exam. Baseline self-report and functional performance and quality of life testing will be conducted using the subject’s usual prosthetic device. (See Standardized Outcome Measures section). If the subject does not have a prosthetic limb, all applicable baseline testing will be performed without a device.

Pre-prosthetic Training

The study therapist will evaluate the subjects’ strength and ROM and prescribe a home exercise program to address any impairment. In addition, all appropriate subjects will be instructed in a home-based rotator cuff strengthening program to prevent excessive muscle soreness due to use of the DEKA Arm.

Part A - Visit 2

Baseline Retesting

This visit will take place within 1 week of Visit 1. It will involve repeat administration of the self-report and functional performance testing administered during Visit 1. This information will be used to help us to determine the stability of the baseline test scores.

Part A - Home Usage Monitoring

Subjects will be asked to maintain daily logs detailing usage of their current prosthesis over a period of two

weeks. Additionally, subjects may be asked to wear an activity monitor for prosthetic use (the exact device would be determined through pilot work.) This data will be collected prior to initiation of in-lab training with the DEKA Arm. These measures of existing prosthetic activity will be compared with those of home use of DEKA Arm to determine the extent of adoption. Subjects who fail to complete the home use monitoring portion of the study may be offered a second chance to complete the at-home log in a new two week time period. Subjects will be re-instructed on the use of the diary. Those who fail to complete study logs a second time will be terminated from the study and will not be fit with a DEKA Arm. Non-prosthetic users will be exempted from this requirement.

Part A – Visits 3-8

Fabrication of the prosthetic socket and set-up of prosthetic controls

After baseline data collection (Visits 1 and 2) are completed, the prosthetist will begin fitting a custom socket and setting up the system controls. Subjects will have their prosthetic fitting, fabrication and systems set-up done at the participating VA site where they were screened. In some cases, the study lead prosthetist may travel to the participating site to provide direct supervision during the fitting and set-up. For all subjects, video and telephone conferencing with the study's lead prosthetist, prosthetic consultant and DEKA engineers will take place as needed to troubleshoot concerns about socket fit and fabrication and controls set-up. All videoconferencing will take place using secure VA video equipment hosted and maintained by VA OI&T. We recognize that there is the possibility that a satisfactory check socket will not be able to be fabricated without direct supervision despite video or in-person prosthetic consultation. Because, testing and training activities with the DEKA Arm cannot commence without a satisfactory socket fit, if this should occur, then study activities may be delayed until a supervisory visit from the study prosthetic team can be arranged.

We expect that the process of socket fabrication and determination of control set-up will take up to 6 sessions which last from 60 minutes to four hours each depending upon the complexity of the case. However, if the prosthetist agrees and the subject consents and their schedules allow, the duration of visits will be extended beyond four hours to accommodate patient preferences. We expect that each subject will require 1-6 check sockets prior to fabrication of the definitive socket.

During prosthetic fit and set-up visits the subject will be familiarized with the arm controls using the Virtual Reality Environment (VRE).The VRE is an interactive computer software program which allows the subject to practice controlling the components of the arm without wearing the prosthetic limb. Subjects using the RC and HC arms will have between 30 minutes and 2 hours of VRE time. The time allotted to VRE training for RC and HC will be determined, by the site therapist. Subjects using the SC arm will receive 4 hours of VRE training.

During fitting and VRE activities, subjects will be provided with regular breaks as needed. Long prosthetic fitting sessions would involve substantial "down time" when the subject is resting or may be doing the VRE training and the socket is being fabricated in the laboratory. During fitting, the subject will have multiple standard impressions taken of their residual limb and torso using a plaster bandage method. A stockinet and a barrier will be placed over the subject's skin. Clothes will be covered to minimize the possibility of staining by the plaster. The impressions will be used to fabricate an appropriate socket with a harness as needed. Measurements of soft tissue compliance or stiffness at multiple locations on the residual limb and torso will be taken.

The subject will be offered a variety of control options and the final decision as to the control scheme will be a collaborative decision-making process between the subject and the prosthetist. Subjects enrolling in the study phase evaluating use of the DEKA Arm with pattern recognition (Coapt) control, are required to use this control scheme as part of their control option. Whenever possible, the occupational therapist will be present during the systems controls set-up process so as to provide additional input into the decision-making process. Prosthetic controls may include surface EMG electrodes and combine the use of touch pads, switches, cables, inertial measurement units (IMUs) and other input devices that track or sense movements, such as foot pads, as well as tactors that provide mechanical stimulation to the skin surface related to activity or contact pressure.

Special Note: The Rivermead Extended Activities Index (REAI) may be completed during 2 consecutive visits

between Visits 1 and 4. Administration must take place between 1 day and 1 week apart. Repeat administration of this instrument will be used to help us to determine the stability of the instrument.

Part A - Training Visits

Once prosthetic fit, set-up and necessary VRE training is complete, subjects will be trained in the use of the DEKA Arm by the study therapist. Each subject will participate in an average of 18 training sessions. The actual number of training sessions may vary from a minimum of 5 for TR amputees to a high of 25 for amputees using the powered shoulder. The exact number of 2-hour training visits will be determined by the site therapist based upon the speed of the subject's acquisition of skills and knowledge needed to use the arm appropriately and safely. We expect that subjects who have used the DEKA Arm in prior VA studies will already be acquainted with the basic functions and controls of the arm and thus will require fewer visits, and subjects who are new to the DEKA Arm as well as those who are powered shoulder users will require more visits. We will cap the number of training visits at 20 (40 hours of training) for TR/TH users and 25 visits (50 hours of training) for SD users.

Training session visits will last a minimum of 2 hours and could last as long as 4 hours (time estimate includes time for donning and doffing the arm, regular breaks, adjusting the system controls and handling other technical matters). If study staff encounter extensive technical difficulties, a session may be extended to accommodate teleconferences to troubleshoot and resolve the technical problems. Subjects will be able to return on another day to complete the session if they are not able to stay for the duration of this time. Five minute rest breaks will be provided every 20-30 minutes. Training activities will include: strengthening and ROM, a review of control sites, repetition of various drills to reinforce the patterns required for prosthetic utilization and a review of "new" prosthetic terminology relating to the DEKA Arm. Prosthetic use training will begin with reinforcement of prosthetic control patterns of motions, proceed to simple grasp and release activities, performance of patient requested activities, and progress to more complex utilization of the prosthesis in unilateral and bilateral tasks, and ultimately lead to supervised community based outings.

Part A - Advanced Training Visits

A minimum of 3 community outings will be conducted towards the end of the training visits. The purpose of these outings is to allow the subject to experience using the DEKA Arm in a public setting; the environment may be noisy or distracting. These outings will be scheduled when the therapist considers the subject adept enough to utilize the arm in a public setting with only minimal coaching and supervision. Key supervised outings will include at least one of each of the following types of activities: 1) eating a meal in public, such as at the cafeteria or a local restaurant, 2) riding in a car (as a passenger) or on some form of public transportation, 3) shopping - entering a store, selecting and carrying purchases and paying for items.

Part A - Testing Visits

All subjects will repeat the baseline battery of tests after the completion of the initial 5 visits of training, and again after training is deemed complete. The plan for data collection at these visits is shown in Table 6. At the end of study testing session subjects will be asked to provide information about their impressions of the arm, the socket, their training experience, readiness for home usage as well as history of falls during the previous month.

Part A - Preliminary Determination of Appropriateness for Unsupervised, Home Device Use

Determination of appropriateness for unsupervised, home device use will be made by the therapist when the therapist thinks that the subject has mastered the knowledge and skills necessary for appropriate home usage, or after completion of the maximally allowable number of study training visits. Therapists will utilize criteria relating to skillfulness of prosthetic use, independence in daily device maintenance and storage and safety awareness. (See Appendix 3: DEKA Arm Do's and Don'ts and Appendix 4: Assessment of Skill and Safety in Device Operation.)

Part B Procedures

Part B - Selection and Consent for Home Study

Subjects who complete all Part A study activities and who are determined to be appropriate for unsupervised home device use will be invited to participate in the Take Home Portion of the study (Part B). Subjects who agree to participate will sign new informed consent forms that explain all study procedures, risks and benefits

Part B - Home Visit

The purpose of the home visit is to determine suitability of the home environment for safe home use and to identify any potential hazards in the subject's home environment which may make home use ill-advised. Two trained study staff members will visit the subject at home to conduct an assessment of the home environment. Elements of the home safety assessment are detailed in Appendix 2. At the completion of the home study assessment, the site PI in conjunction with Dr. Resnik will determine whether or not the subject's home environment is safe and appropriate for unsupervised home device use. This visit may take place over two days to allow time for the study staff to discuss the visit with Dr. Resnik and to deliver the Arm on the second day. During the home visit the study staff member will walk through the subject's home to determine adequacy of plumbing, electricity, and note general cleanliness, presence of pets, and need for major repairs. The staff will identify potential hazards and will discuss these potential hazards with the subject. Together, the staff will identify the location where the subject plans to store and electrically charge the investigational prosthesis. Study staff will take photographs of the subject's home to provide detail and documentation of the home environment. The subject will be asked to invite their emergency contact person and their caretaker if they require one, to be present during this visit. If the study staff identify any immediate safety concerns or recommendations for enhancing the subject's independence in their home environment they will make appropriate referrals and provide additional information.

Part B - Home Usage Monitoring

Prior to sending the subject home with the DEKA Arm the study staff will review all instructional material and make sure that the subject has correct contact information for study personnel. The subject will be informed about all potential hazards of the investigational prosthesis and how to avoid these hazards (see Appendix 1) and agree to follow the instructions and restrictions given to them by the study staff. If needed, a caregiver/companion will be been trained on the use, maintenance, shipping and potential hazards of the investigational prosthesis. Subjects will be provided with 24-hour contact information in the event of an emergency related to use of the DEKA Arm.

Periodic phone calls to the subject at home will be made during the home usage portion of the study. The frequency of these calls will be no less than 1x/week, but may be increased at the discretion of the study staff if there are concerns about compliance or the home environment.

During the first month of home use of the DEKA Arm subjects will be asked to wear and use the arm for a minimum of 2 hours per day. Our expectation is that this requirement will help establish a pattern of regular usage. After 1 month subjects will be instructed to wear and use the DEKA Arm as much and as often as they wish. Throughout Part B subjects will be asked to maintain daily logs detailing use of the DEKA Arm over the course of Part B participation. These daily logs will be mailed to the study staff weekly.

At the monthly in-person study visits, engineering logs will be downloaded from the DEKA Arm and sent to DEKA for data extraction. This technical data will consist of event logs and usage statistics. The event logs consist of time stamped lists of selected internal inter-processor messages. These logs are used to monitor system performance and assist in trouble shooting technical problems. The engineering usage statistics data files consist of histogram data of position/speed/current for each motor drive in the system, as well as histograms of battery parameters such as voltage and current. DEKA will provide the Principal Investigator with usage summaries for each two week period. These will be compared to the subject's self-report.

Part B - Biweekly Study Visits:

Reassessments of self-reported quality of life, prosthetic satisfaction and function will be conducted on a biweekly basis. These visits will occur either by telephone, videoconference, or in-person.

Part B – In-person Visits:

In-person visits will occur at least monthly (and may overlap with the biweekly visit). Reassessments of dexterity and function (measured by performance based tests) will be conducted. Subjects will complete surveys that ask about their impressions of the arm, concerns about the arm, prosthetic comfort and fit, and training. In-person visits will be videotaped to provide qualitative data on functional performance over time. More frequent in-person visits will be scheduled as necessary to address any concerns about arm operation or use.

Prior to the start of Part and at all in-person study visits, Arm Usage Data (engineering logs) will be downloaded from the DEKA Arm by the study site and sent to PVAMC for data extraction.

Part B - Final Study Assessment

The final study visit will include reassessment of all outcome measures tested at baseline (except neuropsychological tests), administration of a participant survey and/or semi-guided interview asking about the experience of home use of the DEKA Arm, the added benefits, challenges, and maintenance needs. In addition, subjects will be asked about their history of falls during the previous month.

Part A and B Additional Considerations

Poor socket fit

In the event that a problem with the definitive socket fit and comfort occurs during the study, the prosthetist may need to fit and fabricate a new socket. This could potentially require up to an additional 14 visits to fit and check the new socket and would only be done if the subject agrees and can accommodate the scheduling of additional visits.

Malfunction of DEKA Arm System

In the event that the DEKA Arm system malfunctions in any manner, subjects will be instructed to contact study staff within 24 hours. If necessary, the study staff will make the determination to put study activities on hold and will consult with DEKA systems engineers. If the problem is not resolvable remotely, the arm will be shipped back to DEKA and repaired. The repaired arm or a new arm will be returned to the study site and study activities will resume. Subjects will not contact DEKA engineers directly for troubleshooting or repairs.

Neuropsychological and other health concerns

Subjects will undergo medical screening prior to enrollment in the study. If, during the course of study activities, the study staff suspect any undiagnosed neurocognitive deficits that were not detected upon screening or if any other health problems arise, subject will be referred for further evaluation outside of the study.

Transition to Part B

There may be instances where participants need to come into the lab for additional prosthetic fitting or training visits after completing Part A and before beginning Part B to ensure that the prosthetic socket fits well, the DEKA arm and controls are working correctly and/or that the subject has retained the skills gained in Part A. These visits may be needed if the subject gains or loses weight, if a change to the controls configuration is needed for any reason or if a significant amount of time has passed since completing Part A (>4 weeks). The Principal Investigator and study staff will consult to determine the best plan of visits on a subject-by-subject basis.

Some subjects, after completing Part A at one site may be invited to continue Part B at a different site. In this case, subjects would make an in-person visit to the new site to meet the staff and have their socket and DEKA Arm function inspected prior to their home visit. These subjects would consent to Part B at the new site and information about them will be shared with the study team. This transition may be needed as the study terminates at 2 sites but continues at a 3rd site.

Study Data Sources

We will use standardized outcome measures collected at key intervals over time to examine outcomes of pain, dexterity, functional activities, prosthetic satisfaction and quality of life (see Appendix 5). Most of these measures were utilized in the VA Study to Optimize the DEKA Arm. Additionally, we will collect qualitative data from home safety assessments (Appendix 2), video logs, surveys, interviews and prosthetic usage journals. Each data type is described in the section below. The proposed time frame of data collection is shown in Table 6 below.

	**PART A**				**PART B**				
	Current Prosthesis		DEKA Arm		DEKA Arm				
	**Baseline Visits 1 &2**	**Prior to DEKA Arm training**	**Visit 14**	**End of Part A**	**Home Visit**	**Weekly**	**Biweekly**	**Monthly**	**End of Part B**
**Outcome Measures**									
Neuropsychological testing	X								
Myosite screening	X								
Pain	X		X	X			X	X	X
Dexterity	X		X	X				X	X
Self-reported Functional Activities	X		X	X			X		X
Prosthetic Satisfaction	X		X	X			X		X
Performance based Functional Activities	X		X	X				X	
Quality of Life	X		X	X			X		X
Community Integration	X		X	X			X		X
**Other Data Sources**									
Home safety assessment					X				
Weekly telephone call						X			
Video logs of performance									
Surveys of patient			X	X				X	X
Guided interviews				X					X
Prosthetic usage journals		X				X			
Prosthetic usage technical data		X						X	
Appropriateness for Home Use				X					
Final Determination of Appropriateness for Home Use					X				
Estimated time to complete assessment	2 hours	10 min	1.5 hours	2 hours	1. 5 hrs	10 min	30 min	1.5 hours	2 hours

Table 6. Schedule of data collection plan

Standardized Outcome Measures

Neuropsychological Testing

All subjects will complete a brief battery of widely-used, standardized, clinical neuropsychological tests at baseline to determine the association between cognitive function and success of the DEKA arm intervention. The battery is designed to assess all major cognitive domains with emphasis on executive function. It is designed to be brief (approximately 35 minutes), easily and reliably administered, and amenable to completion by participants with upper extremity amputation. The battery is comprised of following: Color-Word Interference and Verbal Fluency Tests from the Delis-Kaplan Executive Function System (D-KEFS),[12] Judgment of Line Orientation (JLO),[13] Digit Span and Letter-Number Sequencing subtests from the Wechsler Adult Intelligence Scale – IV (WAIS-IV),[14] oral versions of the Trail Making Test parts A & B,[15] Symbol-Digit Modalities Test,[16] Shape Learning and Shape Recall items from the Screening Module of the Neuropsychological Assessment Battery (NAB),[17] and several components of the Repeatable Battery for the Assessment of Neuropsychological Status (RBANS).[18, 19] Each of these assessment tools is described briefly below. We have determined that total administration time for this battery of tests is approximately 45-55 minutes.

D-KEFS Color-Word Interference Test[12]: This test measures the examinee's ability to inhibit an overlearned (or prepotent) verbal response (i.e., reading a color word aloud) in favor of an alternative response (i.e., naming the color of ink the word is printed in). The Color-Word Interference Test has four parts each involving a 5 X 10 stimulus array. Practice is allowed for each part. First, examinees are presented with a card of colored squares (blue, green, and red only) and are asked to name the colors as quickly as they can (90 second limit). Second, they are asked to read aloud an array of color words (blue, green, and red only) as quickly as possible (90 seconds). The third array (interference condition) contains the words blue, green, and red printed in an incongruous color ink (e.g., "blue" printed in red or green) the correct response is the color of the ink (180 seconds). The fourth condition adds a cognitive set-switching component. Here, the task is the same as in the third condition except some words are surrounded by a box, in which case the correct response is to read the word (180 seconds). The examiner records the completion time and number of errors.

D-KEFS Verbal Fluency Test: This test assesses the individuals' ability to generate words fluently in a difficult phonemic format (Letter Fluency) in comparison to an overlearned format (Category Fluency) and to a cognitive set-switching condition (Category Switching). The test has three conditions. The first condition, Letter Fluency, requires examinees to say words that begin with a specified letter as quickly as possible in 60 seconds. Three trials are administered, each using a different letter. In the second condition, Category Fluency, examinees say words that belong to a specified semantic category (e.g., animals) as quickly as possible in 60 seconds. Two trials are given, each utilizing a different semantic category. In the third condition, Category Switching, examinees say words from two different semantic categories (alternating between them) as quickly as possible in 60 seconds.

Judgment of Line Orientation (JLO) [13]: This test of visuospatial processing assesses the ability to estimate angles between line segments. Examinees are presented with two cards: a stimulus card with two angled, non-intersecting line segments; and a response card with a semi-circular array of 11 numbered lines drawn at 18-degree intervals. The examinee is asked is to select two lines from the array that form the same angle as line segments on the stimulus card. Practice items are provided. There are 30 items; we will use a shortened (15-item) version.

WAIS-IV Digit Span [14]: This is a test of basic and complex verbal attention (working memory). There are three components to this test. All components involve the examiner reading aloud a string of digits in and the examinee repeats them according to specific rules. In the first component "digits forward," the examinee repeats the items in the same order the examiner read them. In the second component, "digits backward," the examinee repeats the digits in reverse order. In the third component, "digits sequencing," the examinee repeats the digits in ascending order. There are 10 items each consisting of two trials. String length increases across items 1 through 10. The test continues until the examinee fails all three trials of a particular item.

WAIS-IV Letter-Number Sequencing [14]: In this test of working memory ability the examiner reads aloud a string of alternating numbers and letters (e.g., 4-K-2-Q-7-M). The examinee is required to repeat back the numbers in ascending order and the letters in alphabetical order. There are 10 items each consisting of three trials. String length increases across items 1 through 10. The test continues until the examinee fails all three trials of a particular item. Practice items are provided.

RBANS[18, 19]: The RBANS is a brief battery of 12 neuropsychological subtests that have been used to assess cognitive function in a wide variety of conditions including those with dementia, stroke, multiple sclerosis, and head injury. We will use the following components of the RBANS: Picture Naming (assesses confrontation naming); List Learning, Recall, and Recognition (assesses non-contextual verbal learning and recall); and Story Learning and Recall (assesses contextual verbal learning and recall). Delay times for List Recall and Story Recall will be set to approximately 10 minutes.

Trail Making Test part B (oral): This is a test of cognitive set-switching involving alphanumeric sequencing.[9] We will use an oral version of this test rather than the standardized pencil-and-paper test.[15] The examinee counts as follows "1-a, 2-b, 3-c . . . 12-l". The examiner records completion time and errors.

NAB Shape Learning and Shape Recall (Screening Module) [17]: In this test of visual learning and memory, examinees are shown a series of five abstract shapes, one at a time, for 5 seconds each. This is followed by the Immediate Recognition trial in which the examinee is shown a series of five 2 x 2 arrays. Each array contains one of the five shapes learned earlier (target) and three similar shapes (distracters). The examinee is asked to identify the target. We will modify this task by having examinees indicate their selection verbally (i.e., "upper right") rather than pointing. The Delayed Recognition trial is administered approximately 10 minutes later. The score is the number of items correctly identified in the recognition trials.

Symbol-Digit Modalities Test[16]: In this widely used test of mental processing speed involving digit-symbol matching. Examinees are presented with a sheet of paper. The top of the page shows a "key" showing the digits 1-9 each paired with an abstract shape. The remainder of the page consists of rows the symbols in mixed order. The examinee is asked to write or state the number that should be matched with the symbol based on the key (which remains in plain view at all times) and to complete as many items as possible correctly in 90 seconds.

Pain

Wong-Baker FACES Pain Rating Scale (FACES)[20]: The FACES is a commonly used 6-point pain scale that utilizes faces to indicate the intensity of pain. The subject is asked to choose the face that best describes how he/she is feeling.

Dexterity

Jebsen-Taylor Hand Function Test (JTHFT) [2]: This seven-part test evaluates the time needed to perform 7 hand-related tasks including: 1) printing a 24-letter, third-grade reading difficulty sentence, 2) turning over 7.6 x 12.7 cm (3x5") cards in simulated page turning, 3) picking up small common objects including pennies, paper clips, bottle caps and placing them in a container, 4) stacking checkers, 5) simulated feeding, 6) moving large empty cans and 7) moving large 1 lb. cans. The subtests are scored by recording the number of seconds required to complete each task. Additionally, we will count the number of sub-activities completed within the allotted two minutes. Increased time to complete the subtest is related to decreased function of the hand. The JTHFT will be administered with the subject seated on an 18 inch stool in front of a table of 30 inches in height. Only the involved side (i.e. prosthetic side) will be tested. Subjects are limited to two minutes to complete each sub-test. The entire test takes about 15 minutes to complete. Prior studies have reported excellent test-retest reliability.[21] This test has been widely used as an outcome measure of upper limb function in studies of prosthetics,[22] orthotics,[23] wrist arthrodesis,[24] stroke,[25-28] brain injury,[29] arthritis,[30, 31] and nerve injury.[32] Normative values for time-based adults have been published.[2]

Functional activities

Performance Based Functional Activities

University of New Brunswick Test of Prosthetic Function (UNB) [33]: The UNB test, one of the best known measures in the upper extremity prosthetics, was originally developed for children.[34] However, others have used the age-appropriate activities from the UNB test as a measure of prosthetic function in upper limb prosthetic training for adults who are not amputees.[35] Inter-rater reliability of the rating scale in a pediatric amputee population has been reported as 0.73-0.91. This test will be used for unilateral amputees only. We will use one of the subtests of activities designed for ages 11-13 year olds, which include activities that are appropriate for adults. The subtest we will use includes the following activities: wrapping a parcel, sewing a button on cloth, cutting meat, drying dishes, sweeping floor, and using a dustpan and brush. No instructions will be given regarding the use of the prosthesis; instead the subject will be directed to do the activity. Performance on the UNB test will be videotaped and the test scored from the video by two VA Credentialed independent raters who are trained in the UNB scoring system. For each of 10 tasks, the amputee is rated on a 5-point scale of 0-4 for dual functions: spontaneity of prosthetic function and skill of prosthetic function. Spontaneity measures the extent to which the amputee has incorporated the prosthesis into his or her body image and measures the tendency to use the prosthesis to assist with the task. Skill measures the dexterity with which the prosthesis is used. It includes the ability to open and close the terminal device to grasp and release objects with confidence, speed and consistency and maintain grasp without letting go accidentally, and apply correct amount of pressure. Spontaneity is rated first, then skill.

Activities Measure for Upper Limb Amputees (AM-ULA): The AM-ULA is an 18-item measure that assesses key elements of functional performance with a prosthesis: the ability of the amputee to complete daily activities, the speed of the performance, the movement quality, skillfulness of prosthetic use and independence. The AM-ULA is a new observational outcome measure of activity performance developed during the VA Study to Optimize the DEKA Arm to address the need for a validated functional activity measure for adults with upper limb amputation. It was implemented part-way through the Gen 2 protocol. The 18 items of the test include: brush/comb hair, put on a T-shirt, remove a T-shirt, button shirt, zip a jacket, put on socks, tie shoe laces, drink from a paper cup, use a fork, use a spoon, pour from a 12 oz can, write the word "LETTER" legibly, use a pair of scissors, turn around doorknob, dial a touch tone phone, use a hammer and nail, fold a bath towel, and reach overhead and grasp an object. Each item of the AM-ULA test was videotaped and the performance was independently scored by two certified hand therapist raters who were trained in the scoring criteria and blinded to the subject's visit number. Extensive psychometric evaluation of the new measure was conducted examining test-retest reliability, inter-rater reliability, and internal consistency. We then chose only those items that had at least moderate (0.5 or above) test-retest and inter-rater reliability to include in the final version of the activity measure. To validate the new measure we used ANOVAs to compare scores at the first testing session (Visit 1) for subjects by level of amputation, expecting that subjects with more proximal levels of amputation would have lower (worse) scores than those with more distal (i.e. transradial) amputations. We also used ANOVAs to compare mean scores (mean of independent raters 1 and 2) for each activity item by amputation level. Analysis showed that the AM-ULA had excellent test-retest reliability, inter-rater reliability and internal consistency. Scores of transradial amputees were significantly better than those of transhumeral and shoulder level amputees.

Extended Activities Measure: The Extended Activities measure is an observational measure of ability to complete specific activities. This assessment tool was implemented for the Gen 3 portion of the study. The eleven activities included in this measure were: tuck shirt in pants, pull down pants, reach a container on a high shelf, open a water bottle, lift a 20 lb box from the floor, remove a wallet from the back pants pocket, replace the wallet in the back pocket, take a gallon jug of water from the refrigerator, open and pour water from a gallon jug, pick up an egg from a carton, and carry a 20 lb bag. Therapists used a standardized set of instructions to administer this test. All activities were graded by the site therapist as "0" unable to complete, or "1" can complete. A standardized definition of each activity was used in the grading.

Rivermead Extended Activities Index (REAI): The REAI is an Instrumental Activities of Daily Living (IADL) measure that was developed to measure functioning in the stroke population, many of whom have upper limb involvement (Whiting and Lincoln 1980). Several studies support the reliability and inter-rater reliability of the REAI. Test retest reliability for persons post CVA was reported to be 0 .95. -0.97 (Whiting and Lincoln 1980; Rossier, Wade et al. 2001). The REAI was validated for persons with stroke and elderly persons (Whiting and Lincoln 1980; Lincoln and Edmans 1990). The REAI is a performance based measured that is scored on a three-point scale: 1, dependent; 2, independent but requires verbal supervision; and 3, independent. It has been used in young stroke patients (<65 years) and elderly stroke patients (>65 years) (Hamrin 1982). We will also add a timed element to the testing procedures.

Self-reported Functional Activities

Upper Extremity Functional Scale (UEFS) from the Orthotics and Prosthetics Users Survey (OPUS): [6, 7]
The UEFS is a self-report measure developed for use with upper limb adult amputees. UEFS items ask clients to evaluate the ease of performing 23 activities including self-care and instrumental daily living tasks using a 5-point scale from “1” very easy to “5” cannot perform. Items include activities varying from washing, buttoning shirt, tying shoe laces, using fork or spoon, and writing name, to donning and doffing the prosthesis. All activities on the UEFS test will be included as part of the standardized training protocol for the study. Preliminary analyses of the UEFS were done using a sample of amputees in Slovenia and the Slavic version of the UEFS. We will supplement the items on the UEFS with up to a dozen other functional activities identified as important for daily living.

The Patient Specific Functional Scale (PSFS): The PSFS is a patient-specific outcome measure to assess functional status.[5] Patients are asked to identify up to five activities that they have difficulty performing due to their condition and then rate the amount of limitation they have in performing these activities on a scale of 0 to 10 with 0 being unable to perform the activity and 10 being able to perform the activity with no problem. Individual items are scored separately. The PSFS has been shown to be valid and responsive to change for patients with neck pain, cervical radiculopathy, knee pain and low back pain.[37, 38] Its use with upper limb amputees has not been previously explored.

QuickDASH Outcome Measure: The QuickDASH is an 11 item self-report measure developed from the original 30-item Disabilities of the Arm Shoulder and Hand Measure (DASH). Each scale item has 5 response options ranging from 0 (no disability) to 100 (most severe disability). At least 10 of the 11 items must be completed for a score to be calculated. The assigned values for all completed responses are summed and averaged, producing a score out of five. This value is then transformed to a score out of 100 by subtracting one and multiplying by 25. A higher score indicates greater disability.

Prosthetic satisfaction

Trinity Amputations and Prosthetics Experience Scale (TAPES): The TAPES was originally developed for people with lower limb amputations, but has recently been used for persons with acquired upper limb amputations.[8] The TAPES assesses three primary areas: psychosocial adjustment, activity restriction and satisfaction with the prosthesis. We will use the 10 item scale related to prosthetic satisfaction which include questions about extent of satisfaction regarding functional characteristics of the artificial limb: reliability, comfort, fit, and overall satisfaction, contentment with cosmetic characteristics of the device.[8] Each item is rated on a 5-point scale from very dissatisfied to very satisfied. Cronbach alpha (internal consistency) for the prosthetic satisfaction scale for upper limb amputees has been reported as 0.94.[8]

Quality of life

SF-36 V: Four scales of the SF-36V (Veterans version) will be used to assess quality of life. The Role Physical Scale is a 4-item subscale that measures difficulty with role function in work or daily activities attributable to physical health problems. [39] The 3-item Role Emotional subscale measures difficulty with role function in work or daily activities attributable to mental health problems. Finally the 2-item Social Functioning subscale measures interference with social activities related to physical and emotional problems. [39] The Physical

Functioning Subscale (PF-10) is a 10-item subscale of the SF-36 that measures difficulty with performance of physical activities.[39]

Quality of Life Scale (QOL): The QOL consists of 16 questions that assess satisfaction with independent living and self-care activities. [40]

The Community Reintegration of Service Members Computer Adaptive Test (CRIS-CAT): The CRIS measures community reintegration of veterans, using the construction of participation as defined by the WHO's International Classification of Health and Functioning. The CRIS-CAT, developed with funding from VA HSR&D, was recently developed from the CRIS. [41] It consists of three scales which assess three different dimensions of community integration, extent and frequency, perceived limitations, and satisfaction. CATs for all three scales require 10-20 items to achieve accuracy above .88.

Other Data Sources

Home Usage Logs - Part A: Subjects will be asked to maintain home usage logs which document the number of hours per day that they wore their existing prosthesis, and the estimated number of hours that they actually utilized the device. They will also be asked to identify the major types of activities that they performed using their prosthesis by checking off Yes/No to major categories of use including: household chores, driving, cooking, eating, recreation, working. Usage logs will also provide space for additional comments.

Home Usage Logs - Part B: We expect that subjects will utilize both their existing prostheses and the DEKA Arm during the home study portion of the trial. Thus, home usage logs for Part B while similar to those of Part A, will also ask specifically about the use of the DEKA Arm.

Activity monitoring/engineering logs: DEKA engineering data files consist of histogram data of position/speed/current for each motor drive in the system, as well as histograms of battery parameters (i.e. voltage and current.) DEKA will provide the PI with usage summaries for each two week period.

Open Ended Survey questions: Study-specific surveys will contain both open-ended questions as well as quantitative scales specific to use of the Gen 3 DEKA Arm. Open-ended questions will ask about the subject's overall impression of the DEKA Arm, the experience of using the arm at home, and will ask the subject to compare the DEKA Arm to current prosthetic device.

Semi-guided interviews: After the last visit, subjects may participate in a semi-structured interview administered by the study therapist or research assistant and will be audio recorded. During these interviews subjects will be asked to expand on the questions asked in the open ended survey questions, to talk about the DEKA Arm and their lifestyle. See Appendix for examples of semi-guided interview questions.

Audio Logs: At each session the subject and study staff will be provided with digital hand held micro recorders and asked to record comments for the investigative team before, during, or after their scheduled visits. Audio files will be uploaded to VA secure Sharepoint or FTP site and will be transcribed verbatim for analysis.

Video Logs: Part A baseline and baseline retesting and reassessment visits will be video recorded. Part B biweekly study visits and the final study visit will be video recorded. Additionally, per request of Dr. Resnik or at the discretion of the site study staff, set-up and training visits may be recorded to capture important milestones or other information. Video logs will be recorded and video data stored on the study secure content server or (if recorded via handheld video camera) will be stored in a restricted folder on the PVAMC P: Drive.

Data Analysis

1. Identify and describe upper limb amputees who would be appropriate candidates for home use of this advanced prosthesis, as well as those who would not be appropriate.

All subjects will participate in Part A of the study, which will involve supervised, in-laboratory training, as well as supervised community based outings. Based upon the findings from Part A, study staff will classify participants as appropriate or not appropriate for participation in Part B (the home usage portion of the study). This

classification will be based upon a specified set of criteria established to determine potential for safe home usage, as well as the completion of all aspects of Part A study activities. Safe home use criteria will include elements such as skillfulness of prosthetic use, independence in daily device maintenance and storage and safety awareness.

We will calculate the percentage of subjects who participate in Part A of the study who are determined to have met the preliminary criteria for home use appropriateness. Based upon our early experience in the VA study to optimize the DEKA Arm, we expect this percentage to be as high as 75-80%. For those subjects who are invited to participate in Part B and consent to do so, we will then calculate the percentage whose home environment is considered appropriate to allow home use.

We will then describe the subjects in each group and examine characteristics of those subjects who were not considered appropriate candidates for home use to identify which, if any factors were predictive. Analysis of variance (ANOVA), t-tests, and χ^2 analyses will be used to compare the appropriate vs. non-appropriate candidates on demographic and clinical variables: age, amputation level, years of education, results of neuropsychological testing. We hypothesize, based upon our preliminary experience, that subjects with long response times for Trail-Making B test may not possess the speed of executive function to enable them to be deemed safe and appropriate home use candidates.

2. Compare the extent of use of the existing prosthesis to that of the DEKA Arm.

Subjects will be asked to maintain daily logs detailing usage of their current prosthesis. Additionally, subjects may be asked to wear an activity monitor for prosthetic use (the exact device would be determined through pilot work). This data will be collected several weeks prior to in-lab training with the DEKA Arm. Quantitative measures of existing prosthetic activity gathered prior to the initiation of the in-laboratory training will be compared with measures taken during the last 2 months of home use of DEKA Arm. We will utilize measures such as logged hours of wear time, hours of use time, and number of times that grips were used (gleaned from automated activities measures and engineering logs). This comparison will allow us to assess adoption of the DEKA Arm in an unsupervised home environment.

3. Quantify the impact of home use of the DEKA Arm on device satisfaction, performance of functional activities and the user's quality of life.

We will use a time series design to examine changes in device satisfaction, performance of functional activities and quality of life throughout the study. Each subject will serve as their own comparison using within subject pre and post intervention measures. To determine whether changes occurred in key dependent variables from baseline with the current prosthesis (Part A) to end of supervised training (Part A) we will use paired t-tests. We will also examine changes in key outcomes from the end of Part A supervised training to the conclusion of the home trial (Part B). Additionally, we will examine the influence of successive usage of the DEKA Arm through linear regression models testing the principle effect of time.

4. ***Quantify the amount and type of technical support and repairs needed during the study, and estimate the number of home study days lost due to service/repair.***

We will gather descriptive data on technical support usage, including number and content of calls and email exchanges with DEKA support, number and type of repairs needed during the study. Study sites will be asked to document each of their contacts with DEKA technical support and forward this information to the PVAMC coordinating site. We will summarize this information across subjects and levels of arm type. This information will be useful in estimating service needs for clinical deployment.

Risks to Subjects

Human Subjects Involvement and Characteristics

Sample

Part A: We expect that we may need to enroll up to 75 amputees across all sites in order to yield complete Part A data on 40 subjects. This is due to anticipated screen failures (estimated at 20%) and the likelihood that some 20% of subjects (based upon prior study experience) who do meet eligiblility criteria will not be able to participate in all research activities. Additionally, we will purposefully sample subjects to insure that we have an even distribution of subjects at each level of DEKA Arm fit during Part B (10 SD, 10 TH, 10 TR).

Part B: We expect that up to 20% of Part A participants may not be considered appropriate for home study usage. Thus, we expect that up to 36 participants who have completed Part A of the study will be invited to participate in the home study portion (Part B) of the study. We will purposefully select subjects who have completed Part A and who are judged to be appropriate candidates for home use (see Part A Preliminary Determination of Appropriateness for Unsupervised, Home Device Use.) Our goal is to have complete data on up to 30 subjects with the Gen 3 DEKA Arm. We plan to include approximately 10 subjects fit at each of the 3 levels of amputation (SD, TH, TR). We estimate that 20% (or 4 subjects) may terminate the study early.

For both parts of the study, an estimated 80% of subjects will be veterans enrolled in the VHA or DoD healthcare systems. Approximately 20% of subjects may be non-veterans whose participation may be needed to meet sampling goals for amputees with higher levels of limb loss, and to insure the inclusion of female amputees. Subjects who have participated in the VA Study to Optimize the DEKA Arm or the VANYHSS Pilot Study to Optimize the DEKA Arm will be eligible to participate in this study.

Sources of Materials

The sources of research material will be tests and questionnaires administered to subjects as well as audio and video recordings all obtained with written informed consent. These data will be used for research purposes only.

Potential Risks

The risks of participation in this study are minimal and are similar to that of being fit for and using a standard commercial battery powered artificial arm.

a) There is a small risk of injury or property damage resulting from improper use of the DEKA Arm under extreme or dangerous conditions.
b) There is a small risk of redness on the skin where the socket is attached. The redness will typically be gone within one day of wearing.
c) The subject may experience some soreness due to the weight of the prosthetic arm and the frequent use of the device during training and testing.
d) There is a risk that the prosthetic arm may possibly bump into the subject’s body or head during function.
e) There is a risk that the battery on the DEKA Arm could overheat and cause burns.
f) There is a risk that wearing the DEKA Arm may cause subjects to experience discomfort in their back and/or shoulder, or that they may experience an increase in pre-existing pain in their back and/or shoulder. To minimize the potential for this, subjects may be asked to stop wearing the arm, and take a break if they begin to experience pain as a result of wearing the DEKA Arm.
g). There is a risk that repeated use of the DEKA foot controls may cause the subject to experience discomfort in their ankle, or that they may experience an increase in pre-existing pain in their ankle.
h) There is some risk of loss of confidentiality in sharing personal experiences or opinions and having performance evaluated through videotape, audiotapes and surveys.

Adequacy of Protection Against Risks

Recruitment and Information Consent

A number of methods will be utilized to recruit subjects into the study. First, clinical staff from study sites will inform potentially eligible subjects about the study and invite them to participate. Second, email notifications regarding the study will be sent periodically to national list-servs for prosthetic and rehabilitation staff. Third, approved flyers and brochures will be distributed at VA/DoD medical centers, private prosthetic treatment centers, and other locations that upper limb amputees might frequent. Fourth, we will distribute press-releases to news groups as well as consumer organizations including the Amputee Coalition of America, Wounded Warriors, and the Disabled Veterans of America. Thus, potential subjects who show an interest in participating can learn more about the study from this informational material. Finally, subjects who participated in earlier VA studies of the DEKA Arm, who consented to be contacted in the future will be informed about the new study and invited to participate.

Consent process

After initial telephone screening to assess eligibility criteria, each potential subject will be scheduled for an in-person assessment which will be held in a private area. Before the assessment begins, the site PI, Research Assistant, or prosthetist will explain the study protocol. Subjects will be asked to make a commitment to be available for all study activities. Individuals will be given adequate time to review and comprehend all information about the study before agreeing to take part, minimizing the possibility of coercion and undue influence.

Protection Against Risk

We will make every effort to protect study participants against these risks as outlined below. The research protocol will be approved by the Institutional Review Boards of each participating VA site and PVAMC before any data collection begins.

a) Risk of injury: Subjects will be provided with a detailed list of prohibited activities and usage advisories (See Appendix 1). Subjects will not be cleared for home use of the DEKA Arm unless they have demonstrated a good comprehension of these safety concerns. In addition, all subjects will have a home safety assessment to insure that they have an adequate environment for using, charging and storing the arm.

b) Risk of skin redness: If redness persists, the Site Principal Investigator and prosthetist will be notified and modifications to the socket will be made as necessary.

c) Risk of soreness: Rest breaks will be provided to minimize this risk.

d) Risk of bump to body or head: The movement of the DEKA Arm will be set slow enough that the subject will be able to reposition the entire prosthesis or move their body to another position to avoid being bumped. During initial in-laboratory training, subjects using powered shoulders will wear safety glasses if appropriate. Furthermore, the prosthetic device will only be used under the supervision of study staff. During initial training, subjects will not be left unattended while wearing the prosthetic device. Only those subjects who have achieved competency with the DEKA Arm (as defined by the study protocol) and are approved by study staff will be allowed to take the arm home for unsupervised use.

e) Risk of battery overheating or burning: To minimize the potential for problems, all batteries are tested by DEKA in a manner that simulates their use in the prosthetic arm. The batteries used in the DEKA Arm are similar to those presently used in commercially available powered prosthetic arms.

f) Risk of loss of confidentiality: Participants must sign an informed consent indicating their willingness to participate in the study, their willingness to be audio taped/recorded and videotaped, their understanding of risks and benefits and their understanding that participation is voluntary. Unless required by law, only the study investigators, members of the investigator's staff and the IRB will have authority to review study records. To protect confidentiality, each subject will be given a unique identification number that will be used in the name field of all study related paperwork. The study numbers will be kept separate from the data and will not contain any personal information. All data regarding subjects (paper and pencil surveys, audiotapes, and data collection forms) will be kept confidential and stored in a secure location (locked filing cabinets) at each

VA/DoD site. Study materials will be stored in accordance with VA Record Control Schedule 10.1 and may also be used for scientific presentation.

All electronic copies of study files will be maintained on the Providence VAMC secure research server in the DEKA-R or DEKA-DATA-R folders. OI&T staff is responsible for all electronic data back-up. Each site will maintain copies of de-identified data in locked filing cabinets within each designated research office.

Potential Benefits of Research to Subjects and Others

Participation in this study will allow subjects to try a new investigational prosthetic device. There is currently no commercial prosthesis which affords the same control features and functional capability as the DEKA Arm. As a result, we expect that some study participants will be able to perform more daily activities with greater ease while using the DEKA Arm as compared to using their existing prosthesis. The study protocol involves intensive training and prosthetic education which may carry over to use of their existing prostheses. This training and education has the potential to result in functional improvements with their current devices.

Importance of Knowledge to be Gained

Information gleaned from this study will be critically important in moving the DEKA Arm closer to commercialization and successful dissemination and deployment within the VA system of healthcare. An important outcome of this study will be the development of clinical guidelines for prescription of the DEKA Arm. Currently, VA Prosthetics and Sensory Aids has few, if any, clinical guidelines to inform prescription of advanced technologies, determine appropriate candidates or readiness for home use of prosthetic devices

Data and Safety Monitoring Plan

A Data Monitoring Committee is not required for this project. The Principal Investigator will have primary responsibility for day-to-day monitoring of the quality of operations in all data collection, data cleaning and transfer activities.

The research protocol will be approved by the Institutional Review Boards of PVAMC, VANYHHS, Tampa VA, and the CFI before any data collection begins.

Serious adverse events and all unexpected adverse events will be assessed continuously and as reported to the PI immediately. Monitoring by the IRB/R&D is conducted at the continuing reviews as scheduled, whenever modification requests are considered, and upon receiving reports of adverse events from the investigator or anyone else.

Other information

Acknowledgment of VA policy to include women and minorities in clinical research

Neither women nor minorities will be discriminated against in the recruitment of subjects for this study. Although the population of veteran upper limb amputees is predominantly Caucasian males, we will make every attempt to include both women and minority subjects in the study.

Financial Information

Subjects will not be charged for any study-related procedures. After the study concludes, the fabricated socket and the DEKA Arm will be returned to the research group. All non-active duty subjects will be paid a stipend of $80 per completed visit which amounts to approximately $25/hour for time spent participating in the in-laboratory training and testing activities. Subjects will be paid $10 per day for in-home usage monitoring. To encourage return of the DEKA Arm, the final payment may be withheld until the arm is returned.

Visits may be split into 2 sessions to accommodate scheduling needs for subject and study staff. In these instances, subjects will be paid an $80 stipend for the overall visit, but provided with travel and meals reimbursement by the day. Additionally, non-active duty subjects will receive a $10 meal allowance for any visits (excluding those that take place at distant sites for prosthetic fitting) that take more than 3.5 hours, and $30 flat travel allowance per day (excluding those days spent away from home) If subjects require more visits,

they will be reimbursed accordingly.

Reimbursement for active-duty participants will be made in accordance with applicable military rules and regulations. Military policy prohibits those who are active duty military from receiving stipends and/or gift cards. Therefore subjects who are active duty military will not be offered stipends for participation. For those not eligible to receive stipends, we may (if the site specific IRB agrees) donate the gift card to that participant's Family Readiness Group (FRG) or other community/social service organization serving wounded warriors.

Special note: Some subjects may be invited to participate in presentations and demonstrations of the DEKA Arm during or following completion of study activities. These Public Relations activities are not considered to be part of study protocol activities. These sessions are optional and subjects will be informed that their participation in current or future study activities will not be impacted in any fashion by their decision to participate in such presentations or not. If the subject agrees to participate they will be asked to sign a separate PR Release form (distributed by Public Affairs). All travel costs associated with such activities will be paid for by the VA.

Special Travel Funds
Additional, supplemental travel funds will be available for use at the discretion of the Study PIs to defray costs of nightly hotel accommodations, travel and per locality based per diem expenses for subjects and caregivers who must travel more than 2 hours (each way) to participate in study activities at the participating VA sites. We expect that up to 50% of subjects at each site will need to travel and stay overnight to participate in study activities.

Reimbursement for Subjects Study Time
Subjects who travel for participation in study activities will be paid $80/day, plus an approved locality based per diem meal allowance during the time they are away. In addition, we will cover costs of hotel expenses during the stay as well as transportation from their home. For subjects who need to travel with a caregiver, we will provide a per diem for the caregiver as well as travel expenses.

Withdrawal from Study
If subjects withdraw from the study, they will be paid for the visits they participated, but not for any additional days.

Withdrawal information
Subjects have the right to withdraw from the study at any time by notifying the Principal Investigator orally or in writing. Subjects can also be withdrawn from the study at the discretion of the site PI due to non-compliance, missed appointments, not following instructions, illnesses or problems that affect participation in the study. If subjects withdraw or are withdrawn from the study, all photographs, audio and/or video recordings up to the point of withdrawal will remain on file for research purposes. Subjects who withdraw will be asked to come to the study site for a final visit and complete all end of study questionnaires and testing. If the subject is unable or unwilling to come to the study site for a final visit and is in possession of the DEKA Arm at the time of withdrawal, they will be required to make arrangements to ship the arm back to the study site. All shipping costs will be covered by the VA.

Termination
Subjects may be terminated from either portion of the study at any time at the discretion of study staff if there are concerns about physical, mental or behavioral health that may impact the wellbeing of the subject or staff.

Data Collection Procedures:
All data for this study are collected at participating study sites using a study code (i.e. subject number) that does not identify the patient. **No primary data is collected by PVAMC (therefore there are no consent forms approved for PVAMC).** All data is transferred via secure VA approved methods to PVAMC study staff for data analysis.

The following types of data are collected for this study:
- paper data
- electronic data
- photograph and video data (note subjects must sign a separate consent for use of Picture/voice).

Data Transmittal Procedures:
Electronic VA sensitive data or information collected as part of this protocol may only be transmitted using VA-approved solutions such as FIPS 140-2 validated encryption. Electronic data sent via regular mail or delivery service, such as on disk, will be encrypted with FIPS 140-2 validated encryption and will be sent via delivery service with a chain of custody.

Data Storage:
All electronic VA sensitive information will be stored on the secure VA server located at:
\\vhaprofpc14\Research\DEKA-R
\\vhaprofpc14\Research\DEKA-DATA-R

All paper VA sensitive information will be stored in the research offices of Linda Resnik in Building 32.
All data collected in connection with this research study will be stored in the VA protected environment. No VA sensitive data will be transmitted outside the VA, with the exception of photographs and videos that are sent to DEKA staff as part of the study CRADA between the VA and DEKA.

Study data will be entered by study staff directly to the Providence VAMC secure research server folders listed above. OI&T staff is responsible for all electronic data back-up.

All mobile devices used as part of this study will be encrypted (FIPS 140-2 validated).
Data collected from this study will be used for research purposes only. No patient identifiable information will be released or published without written permission unless required to do so by law.

Training of Study Staff:
All study staff listed on this project will maintain up to date training for Information Security Awareness and Rules of Behavior. In addition, DEKA staff working on this project take all required research training as required by the VA Business Associates Agreement (BAA). Training certificates for VA and DEKA staff are on file with the PVAMC Research Office.

Data Access:
Only VA study staff listed on the IRB application will be given access to study files (paper, electronic, photo and video). The study PI will terminate access to study records when a user no longer requires access to them.
If there is a theft, loss, or other unauthorized access of study data or storage devices associated with this study, this will be reported according to VA regulations.

Data Analysis:
As data is collected, it is entered into Microsoft excel or word and then transferred to the appropriate statistical programs (STATA, SAS, SPSS, NVivo) for analysis. The PVAMC Research Service maintains the licenses for all listed statistical software.

All data collected as part of this study will be stored and analyzed at the PVAMC. A summary of the data analysis will be shared with DEKA per the VA- DEKA CRADA. This summary will not contain any identifiable data. Only VA non-sensitive data will be transmitted electronically via internet for discussion during study meetings.

Data Removal:
In the event that VA sensitive electronic data requires removal from the VA protected environment, the PI will obtain written approval from the supervisor and concurrence from the ISO and CIO. In addition, the PI will obtain written approval from the supervisor and concurrence from the ISO and PO to physically remove VA

sensitive information in paper format from the VA protected environment in connection with this study.

Other:
Records will be maintained in accordance with the Department of Veterans Affairs Record Control Schedule 10-1. In the event that theft, loss of other unauthorized access of sensitive data or storage devices and non-compliance with security controls occur, study staff has been instructed to follow the Providence VA Medical Center's standard operating procedure on incidence reporting.

Project Management Plan:
This study will be directed by Principal Investigator Linda Resnik, a physical therapist researcher who is based at the Providence VA Medical Center. Dr. Resnik has already assembled a highly qualified team of collaborators across VA and DoD sites, and has a demonstrated track record of multi-site leadership in upper limb prosthetics research. The PVAMC research team is the only team in the country who has direct experience with the DEKA Arm and is well prepared to lead the proposed study. The PVAMC team includes a project manager, data manager, research assistant, qualitative data analysts, and several graduate students. The proposed study will keep this research team intact, utilize study staff at 3 of the 4 data collection sites who participated in the VA Study to Optimize the DEKA Arm, and add up to 2 additional sites in a staggered fashion.

Dr. Resnik's team will be responsible for orienting and training Research Assistants and study staff at all sites. The Study Lead Prosthetist will be responsible for working with DEKA as needed to train any new prosthetic staff added to the study. Dr. Resnik will be responsible for training study therapists. Each site will be staffed by a Site Principal Investigator, at least one prosthetist and therapist (either OT or PT), study physician (who may be the PI), and a research assistant. Some sites may also have additional back-up staff. At the outset of study activities or major changes to the protocol a site visit will be conducted by Dr. Resnik and the Project Manager for in-person orientation and training. Per request of Dr. Resnik, the lead prosthetist may also travel to assist in training team members at sites. The project team will meet weekly through telephone and videoconferencing to discuss project activities. The project team will convene annual for a group meeting. Additionally, group training sessions will be held as needed at the PVAMC or other study locations.

VA RR&D has already made a major investment in purchasing dedicated IT equipment specifically for Dr. Resnik's study of the DEKA Arm. The current study of the DEKA Arm has an established videoconferencing network with secure VA video equipment hosted and maintained by VA OI&T. PVAMC owns 4 Tandberg videoconferencing systems for use at data collection sites, and another 4 systems for service, support and communication. In addition, PVAMC owns a dedicated content server specifically for the VA Study to Optimize the DEKA Arm, and maintains Tandberg service contracts for all equipment. Additionally, PVAMC has an established secure VA Sharepoint as well as a secure FTP site for sharing study documents and files between sites. We expect to utilize this IT network and equipment in the proposed study.

Whenever possible audio wave files, handheld video files and surveys will be uploaded to a study specific VA maintained secure Sharepoint or FTP sites. In the event that this is not possible, all data files will be mailed to PVAMC using a secure courier and tracking number. All video and audio data will be kept on the study's secure content server or on the PVAMC P: drive in a restricted folder. The PVAMC research team will be responsible for timely transcription and data entry. Results of quantitative tests and measures will be scored and analyzed in an ongoing fashion by Dr. Resnik's team.

Duration of the study and timeline

Project activities as shown in the Gannt chart below (Table 7) will commence immediately upon funding and receipt of IRB approval. We anticipate that the length of the data collection period will be a minimum of 36 months and that we will need 6 months for data analysis and dissemination activities.

	Year 1				Year 2				Year 3				Year 4	
	Qtr 1	Qtr 2	Qtr 3	Qtr 4	Qtr 1	Qtr 2	Qtr 3	Qtr 4	Qtr 1	Qtr 2	Qtr 3	Qtr 4	Qtr 1	Qtr 2
IRB approval	x													
Staff training	x													
Part A data collection 3 sites		x	x	x	x	x	x	x	x	x	x	x		
Part B data collection 3 sites			x	x	x	x	x	x	x	x	x	x		
Training new site staff				x	x									
Data collection site 4					x	x	x	x	x	x	x	x		
Data collection site 5						x	x	x	x	x	x	x		
Preliminary data analysis				x	x									
Data analysis											x	x	x	
Dissemination and reporting												x	x	

Table 7. Timeline of Study Activities

References

1. Communication DoREa. User-Friendly Handbook for Mixed Method Evaluation. 1997; www.ehr.nsf.gov/EHR/REC/pubs/NSF97-153?CHAP1.HTM. Accessed 3/21, 2001.
2. Jebsen RH, Taylor N, Trieschmann RB, Trotter MJ, Howard LA. An objective and standardized test of hand function. *Arch Phys Med Rehabil.* Jun 1969;50(6):311-319.
3. Svensson E, Hager-Ross C. Hand function in Charcot Marie Tooth: test retest reliability of some measurements. *Clin Rehabil.* Oct 2006;20(10):896-908.
4. Desrosiers J, Bravo G, Hebert R, Dutil E, Mercier L. Validation of the Box and Block Test as a measure of dexterity of elderly people: reliability, validity, and norms studies. *Arch Phys Med Rehabil.* Jul 1994;75(7):751-755.
5. Stratford P, Gill C, Westaway M, Binkley J. Assessing disability and change on individual patients: a report of a patient specific measure. *Physiotherapy Canada.* 1995;47(4):258-263.
6. Heinemann AW, Bode RK, O'Reilly C. Development and measurement properties of the Orthotics and Prosthetics Users' Survey (OPUS): a comprehensive set of clinical outcome instruments. *Prosthet Orthot Int.* Dec 2003;27(3):191-206.
7. Burger H, Franchignoni F, Heinemann AW, Kotnik S, Giordano A. Validation of the orthotics and prosthetics user survey upper extremity functional status module in people with unilateral upper limb amputation. *J Rehabil Med.* May 2008;40(5):393-399.
8. Desmond DM, MacLachlan M. Factor structure of the Trinity Amputation and Prosthesis Experience Scales (TAPES) with individuals with acquired upper limb amputations. *Am J Phys Med Rehabil.* Jul 2005;84(7):506-513.
9. Reitan R. Validity of the Trail Making Test as an indicator of organic brain damage. *Perceptual and Motor Skills.* 1958;8:271-276.
10. Virzi R. Refining the Test Phase of Usability Evaluation: How Many Subjects is Enough? *Human Factors.* 1992;34(4):457-468.
11. Sawyer D. *Do it By Design: An Introduction to Human Factors in Medical Devices*: U.S. Department of Health and Human Services Public Health Service Food and Drug Administration Center for Devices and Radiological Health;1996.
12. Delis DK, E Kramer, JH Delis-Kaplan Executive Function System (D-KEFS)San Antonio, TX: The Psychological Corporation; 2001.
13. Benton AS, AB Hamsher, K deS Varney, NR Spreen, O Contributions to Neuropsychological AssessmentOrlando, FL: Psychological Assessment Resources; 1994.
14. Wechsler DC, DL Raiford, SE Wechsler Adult Intelligence Scale; Fourth Edition. *Technical and Interpretive Manual.* Vol San Antonio, TX: Pearson; 2008.
15. Abraham E, Axelrod BN, Ricker JH. Application of the oral trail making test to a mixed clinical sample. *Archives of Clinical Neuropsychology.* 1996;11(8):697-701.
16. Smith A. *Symbol digit modalities test*. Los Angeles, CA: Western Psychological Service; 1982.
17. Stern RA, White T, Psychological Assessment Resources Inc. *NAB, neuropsychological assessment battery : administration, scoring, and interpretation manual*. Lutz, FL: Psychological Assessment Resources; 2003.
18. McKay C, Casey JE, Wertheimer J, Fichtenberg NL. Reliability and validity of the RBANS in a traumatic brain injured sample. *Arch Clin Neuropsychol.* Jan 2007;22(1):91-98.
19. Randolph C, Tierney MC, Mohr E, Chase TN. The Repeatable Battery for the Assessment of Neuropsychological Status (RBANS): preliminary clinical validity. *J Clin Exp Neuropsychol.* Jun 1998;20(3):310-319.
20. Stuppy DJ. The Faces Pain Scale: reliability and validity with mature adults. *Appl Nurs Res.* May 1998;11(2):84-89.

21. Hackel ME, Wolfe GA, Bang SM, Canfield JS. Changes in hand function in the aging adult as determined by the Jebsen Test of Hand Function. *Phys Ther.* May 1992;72(5):373-377.
22. Dromerick AW, Schabowsky CN, Holley RJ, Monroe B, Markotic A, Lum PS. Effect of training on upper-extremity prosthetic performance and motor learning: a single-case study. *Arch Phys Med Rehabil.* Jun 2008;89(6):1199-1204.
23. Stern EB, Sines B, Teague TR. Commercial wrist extensor orthoses. Hand function, comfort, and interference across five styles. *J Hand Ther.* Oct-Dec 1994;7(4):237-244.
24. Rayan GM, Brentlinger A, Purnell D, Garcia-Moral CA. Functional assessment of bilateral wrist arthrodeses. *J Hand Surg [Am]*. Nov 1987;12(6):1020-1024.
25. Kraft GH, Fitts SS, Hammond MC. Techniques to improve function of the arm and hand in chronic hemiplegia. *Arch Phys Med Rehabil.* Mar 1992;73(3):220-227.
26. Blennerhassett J, Dite W. Additional task-related practice improves mobility and upper limb function early after stroke: a randomised controlled trial. *Aust J Physiother.* 2004;50(4):219-224.
27. Wu CW, Seo HJ, Cohen LG. Influence of electric somatosensory stimulation on paretic-hand function in chronic stroke. *Arch Phys Med Rehabil.* Mar 2006;87(3):351-357.
28. Wetter S, Poole JL, Haaland KY. Functional implications of ipsilesional motor deficits after unilateral stroke. *Arch Phys Med Rehabil.* Apr 2005;86(4):776-781.
29. Neistadt ME. The effects of different treatment activities on functional fine motor coordination in adults with brain injury. *Am J Occup Ther.* Oct 1994;48(10):877-882.
30. Stern EB, Ytterberg SR, Krug HE, Mahowald ML. Finger dexterity and hand function: effect of three commercial wrist extensor orthoses on patients with rheumatoid arthritis. *Arthritis Care Res.* Jun 1996;9(3):197-205.
31. Stamm T, Mathis M, Aletaha D, Kloppenburg M, Machold K, Smolen J. Mapping hand functioning in hand osteoarthritis: comparing self-report instruments with a comprehensive hand function test. *Arthritis Rheum.* Oct 15 2007;57(7):1230-1237.
32. Amirjani N, Thompson S, Satkunam L, Lobay GL, Chan KM. The impact of ulnar nerve compression at the elbow on the hand function of heavy manual workers. *Neurorehabil Neural Repair.* Jun 2003;17(2):118-123.
33. Wright V. Measurement of Functional Outcome with Individuals who Use Upper Extremity Prosthetic Devices: Current and Future Directions. *Journal of Prosthetics and Orthotics.* 2006 2006;18(2):46-56.
34. Wright FV, Hubbard S, Naumann S, Jutai J. Evaluation of the validity of the prosthetic upper extremity functional index for children. *Arch Phys Med Rehabil.* Apr 2003;84(4):518-527.
35. Lake C. Effects of prosthetic training on upper-extremity prosthesis use. *Journal of Posthetics & Othotics.* 1997;9(1):3-9.
36. Atkins D, Meier R. *Comprehensive management of the upper-limb amputee*. New York: Springer-Verlag; 1989.
37. Chatman AB, Hyams SP, Neel JM, et al. The Patient-Specific Functional Scale: measurement properties in patients with knee dysfunction. *Phys Ther.* Aug 1997;77(8):820-829.
38. Westaway MD, Stratford PW, Binkley JM. The patient-specific functional scale: validation of its use in persons with neck dysfunction. *J Orthop Sports Phys Ther.* May 1998;27(5):331-338.
39. Ware JE, Jr., Snow K, Kozinski M, Gandek B. *SF-36 Health Survey: Manual and Interpretation Guide*. Boston: The Health Institute New England Medical Center; 1993.
40. Burckhardt CS, Anderson KL. The Quality of Life Scale (QOLS): reliability, validity, and utilization. *Health Qual Life Outcomes.* 2003;1:60.
41. Resnik L, Plow M, Jette A. Development of CRIS: measure of community reintegration of injured service members. *J Rehabil Res Dev.* 2009;46(4):469-480.
