# Supplementary material for: EMG pattern recognition compared to foot control of the DEKA Arm
Source: PLoS One. 2018 Oct 18;13(10):e0204854. doi: 10.1371/journal.pone.0204854 (PMC6193636; doi:10.1371/journal.pone.0204854)
Supplement: S3 File — (PDF) [file pone.0204854.s007.pdf]

ClinicalTrials.gov Protocol Registration and Results System (PRS) Receipt
Release Date: July 5, 2017

ClinicalTrials.gov ID: NCT01551420

Study Identification

Unique Protocol ID: A9226-R

Brief Title: Home Study of an Advanced Upper Limb Prosthesis

Official Title: Home Study of an Advanced Upper Limb Prosthesis

Secondary IDs: A0771-R [Grantor or Funder: VA RR&D]

Study Status

Record Verification: July 2017

Overall Status: Recruiting

Study Start: April 1, 2012 [Actual]

Primary Completion: December 29, 2017 [Anticipated]

Study Completion: February 28, 2018 [Anticipated]

Sponsor/Collaborators

Sponsor: VA Office of Research and Development

Responsible Party: Sponsor

Collaborators:

Oversight

U.S. FDA-regulated Drug: No

U.S. FDA-regulated Device: Yes

Unapproved/Uncleared Device: No

Pediatric Postmarket Surveillance: No

U.S. FDA IND/IDE: No

Human Subjects Review: Board Status: Approved
Approval Number: 06/08/2011
Board Name: IRB
Board Affiliation: Providence VA Medical Center
Phone: (401) 273-7100
Email: aising.caffrey@va.gov
Address:

VA Medical Center, Providence
830 Chalkstone Avenue

Providence, RI 02908
Country: USA

Data Monitoring: No

Study Description

Brief Summary: The overall study objective is to examine the feasibility, acceptance, and benefits of home use of an advanced upper limb prosthetic device as well as the logistical support requirements utilized during 3 months of home usage. All participating subjects will enroll in Part A of the study which will involve supervised training. Eligible subjects will be invited to participate in Part B, the home use portion of the study.

Detailed Description: The specific objectives of this study are to:

1. identify and describe upper limb amputees who would be appropriate candidates for home use of this advanced prosthesis as well as those who would not be appropriate.
2. Compare the extent of use of the existing prosthesis to that of the DEKA Arm.and quantify the impact of home use of the DEKA Arm on device satisfaction, performance of functional activities and the user's quality of life.
3. Compare the outcomes of users of pattern recognition to the outcomes of users without
4. quantify the amount and type of technical support and repairs needed during the study, and estimate the number of home study days lost due to service/repair.

All participating subjects will enroll in Part A of the study, which will involve supervised, in-laboratory training, as well as supervised community based outings. Based upon the findings from Part A, study staff will classify participants as appropriate or not appropriate for participation in Part B (the home usage portion of the study). Final determination of appropriateness for home use will be made after a home visit. The home use portion of the study will last for 13 weeks and involve regularly scheduled study visits.

Measures of existing prosthetic activity will be compared with those of home use of the advanced upper limb prosthetic device to determine the extent of adoption. Changes in device satisfaction, performance of functional activities, and quality of life will be evaluated throughout the study. Data on technical support usage will also be gathered.

Conditions

Conditions: Traumatic Amputation of Arm

Keywords: Amputation
Prosthetic Design
Upper Extremity

Study Design

Study Type: Observational

Observational Study Model: Cohort

Time Perspective: Prospective

Biospecimen Retention: None Retained

Biospecimen Description:

Enrollment: 100 [Anticipated]

Number of Groups/Cohorts: 1

Groups and Interventions

Groups/Cohorts	Interventions
Group 1<br>Subjects with upper limb amputation	Device: Advanced upper limb prosthetic device<br>There are three versions of the advanced upper limb prosthetic device: shoulder configuration (SC, also known as shoulder disarticulation (SD)); humeral configuration (HC also known as transhumeral (TH)) and radial configuration (RC also known as transradial (TR)).<br>Other Names:<br>• DEKA Arm, DARPA Arm

Outcome Measures

Primary Outcome Measure:

1. Change in Quality of Life (QOL) scale
   The QOL consists of 16 questions that assess satisfaction with independent living and self-care activities

   [Time Frame: baseline and after 13 weeks of home use]

Secondary Outcome Measure:

2. Home Use Appropriateness
   Performance based criteria (developed for this study) relating to skillfulness of prosthetic use, independence in daily device maintenance and storage, and safety awareness.

   [Time Frame: Up to 6 Weeks of in lab use]
3. Extent of Prosthetic Use
   Daily logs (developed for this study) detailing usage of current prostheses and the advanced prosthetic device.

   [Time Frame: 2 weeks prior to in-laboratory training, and daily throughout the home usage portion of the study]
4. Trinity Amputations and Prosthetics Experience Scale (TAPES)
   The TAPES Satisfaction scale is a 10 item scale which includes questions about extent of satisfaction regarding functional characteristics of the artificial limb: reliability, comfort, fit, and overall satisfaction, contentment with cosmetic characteristics of the device.

   [Time Frame: Baseline, Visit 14, Up to 6 Weeks of in lab use, Bi-Weekly (throughout the home usage portion of the study), After 13 weeks of home use]
5. Activities Measure for Upper limb amputees (AM-ULA)
   The AM-ULA is a new 18 item observational measure that assesses key elements of functional performance with a prosthesis: ability of the amputee to complete daily activities, speed of the performance, movement quality, skillfulness of prosthetic use and independence.

   [Time Frame: Baseline, Visit 14, Up to 6 Weeks of in lab use, Bi-Weekly (throughout the home usage portion of the study), After 13 weeks of home use]
6. SF-36 V

Four scales of the SF-36V will be used to assess quality of life: Role Physical, Role Emotional, Social Functioning, and Physical Functioning

[Time Frame: Baseline, Visit 14, Up to 6 Weeks of in lab use, Bi-Weekly (throughout the home usage portion of the study), After 13 weeks of home use]

7. Neuropsychological Testing Battery
The battery is comprised of following tests: Color-Word Interference and Verbal Fluency from the Delis-Kaplan Executive Function System (D-KEFS), Judgment of Line Orientation (JLO), Digit Span and Letter-Number Sequencing subtests from the Wechsler Adult Intelligence Scale - IV (WAIS-IV), oral versions of the Trail Making Test parts A & B, Symbol-Digit Modalities Test, Shape Learning and Shape Recall items from the Screening Module of the Neuropsychological Assessment Battery (NAB), and several components of the Repeatable Battery for the Assessment of Neuropsychological Status (RBANS).

[Time Frame: Baseline]

8. Wong-Baker FACES Pain Rating Scale (FACES)
The FACES is a 6-point pain scale that utilizes faces to indicate the intensity of pain. The subject is asked to choose the face that best describes how he/she is feeling.

[Time Frame: Baseline, Visit 14, Up to 6 Weeks of in lab use, Bi-Weekly (throughout the home usage portion of the study), After 13 weeks of home use]

9. Jebsen-Taylor Hand Function Test (JTHFT)
This seven-part test evaluates the time needed to perform 7 hand-related tasks including: 1) printing a 24-letter, third-grade reading difficulty sentence, 2) turning over 7.6 x 12.7 cm (3x5") cards in simulated page turning, 3) picking up small common objects including pennies, paper clips, bottle caps and placing them in a container, 4) stacking checkers, 5) simulated feeding, 6) moving large empty cans and 7) moving large 1 lb. cans.

[Time Frame: Baseline, Visit 14, Up to 6 Weeks of in lab use, Monthly (throughout the home usage portion of the study)]

10. University of New Brunswick Test of Prosthetic Function (UNB)
This test will be used for unilateral amputees only. The investigators will use a subtest which includes the following activities: wrapping a parcel, sewing a button on cloth, cutting meat, drying dishes, sweeping floor, and using a dustpan and brush.

[Time Frame: Baseline, Visit 14, Up to 6 Weeks of in lab use, Monthly (throughout the home usage portion of the study)]

11. Adapted Rivermead Extended Activities Index (REAI)
The investigators will utilize selected items from the REAI, a performance based instrumental activity measure that is scored on a three-point scale: 1, dependent; 2, independent but requires verbal supervision; and 3, independent. The investigators will also add a timed element to the testing procedures.

[Time Frame: Baseline, Visit 14, Up to 6 Weeks of in lab use, Monthly (throughout the home usage portion of the study)]

12. Upper Extremity Functional Scale (UEFS) from the Orthotics and Prosthetics Users Survey (OPUS)
The UEFS is a self-report measure developed for use with upper limb adult amputees. It includes 23 activities: self-care, instrumental, and daily living tasks using a 5-point scale from 0- cannot perform the activity to 4-very difficult.

[Time Frame: Visit 14, Up to 6 Weeks of in lab use, Bi-Weekly (throughout the home usage portion of the study), After 13 weeks of home use]

13. The Patient Specific Functional Scale (PSFS)
The PSFS is a patient-specific outcome measure to assess functional status. Subjects identify up to five activities that they have difficulty performing due to their condition and then rate the amount of limitation they have in performing on a scale of 0 to 10 with 0 being unable to perform the activity and 10 being able to perform the activity with no problem.

[Time Frame: Baseline, Visit 14, Up to 6 Weeks of in lab use, Bi-Weekly (throughout the home usage portion of the study), After 13 weeks of home use]

14. The Community Reintegration of Service Members Computer Adaptive Test (CRIS-CAT)
The CRIS measures community reintegration of Veterans, using the construction of participation. It consists of three scales which assess three different dimensions of community integration, extent and frequency, perceived limitations, and satisfaction.

[Time Frame: Baseline, Visit 14, Up to 6 Weeks of in lab use, Bi-Weekly (throughout the home usage portion of the study), After 13 weeks of home use]

Eligibility

Study Population: Males and females with single or bilateral upper limb amputation

Sampling Method: Non-Probability Sample

Minimum Age: 18 Years

Maximum Age:

Sex: All

Gender Based: No

Accepts Healthy Volunteers: No

Criteria: Inclusion Criteria:

Inclusion Criteria (Parts A and B):

- All subjects must be at least 18 years old and have single or bilateral upper limb amputation.
- Subjects must be able to understand the requirements of the study and sign an Informed Consent Form and HIPAA Authorization Form.
- Subjects will include those who are current users of any type of prosthetic device (body powered, electric or hybrid), non-users of devices who have been previously fit with a device, but have chosen not to wear it, as well as new users of devices.
- To participate in the study all subjects must have active control over one or both ankles, OR have an appropriate number of myoelectric and/or other control sites (as determined by the Principal Investigator in conjunction with the research team) to allow adequate prosthetic controls configuration.

Inclusion Criteria Part B only:

- Completion of all Part A study activities.
- Meets all criteria for Preliminary Determination of Appropriateness for Unsupervised, Home Device Use.

Exclusion Criteria:

Exclusion Criteria (Parts A and B):

- Amputees with elbow disarticulation, wrist disarticulation and partial hand amputations will be excluded. *beginning July 1, 2016 amputees with shoulder disarticulaton or forequarter amputation will be excluded.
- Amputees will be excluded if the length of their residual limb would prohibit socket fitting, as determined by the study prosthetist.
- Persons with significant uncorrectable visual deficits that would impair the ability to see the prosthesis and those who have major communication or neurocognitive deficits will be excluded.
- Persons with skin conditions such as burns or poor skin coverage as well as those with severe contractures that prevented prior prosthetic wear will be excluded.
- Persons with electrically controlled medical devices including pacemakers, and implanted defibrillators will be excluded.
- Persons with neuropathy, uncontrolled diabetes, who are receiving dialysis, have insensate feet, severe phantom pain or a history of skin ulcers or any other significant comorbidity which would interfere with the study will be excluded.

- Those with severe circulatory problems including peripheral vascular disease and pitting edema will be excluded.
- Persons with cognitive deficits or mental health problems that would limit their ability to participate fully in the study protocol will be excluded.
- Women who are pregnant or who plan to become pregnant in the near future will also be excluded.
- The investigators will exclude those amputees who work for prosthetic companies that may be considered competitors for the prosthetic arm technology in the future.
- Persons taking medication which poses a risk for operation of heavy equipment will be excluded.

Contacts/Locations

Central Contact Person: Frantzy Acluche, MSc
Telephone: (401) 273-7100 Ext. 6274
Email: Frantzy.Acluche@va.gov

Central Contact Backup: Kate J Barnabe, MHA
Telephone: (401) 273-7100 Ext. 6272
Email: Kate.Barnabe@va.gov

Study Officials: Linda J Resnik, PhD MS
Study Principal Investigator
Providence VA Medical Center, Providence, RI

Locations: United States, Rhode Island
Providence VA Medical Center, Providence, RI
[Recruiting]
Providence, Rhode Island, United States, 02908
Contact: Candace M Shuman 401-273-7100 Ext. 3872
Candace.Shuman@va.gov
Contact: Regina A Correa-Murphy, BS (401) 273-7100 Ext. 3478
Regina.Correa-Murphy@va.gov
Principal Investigator: Linda J Resnik, PhD MS

United States, New York
Manhattan Campus of the VA NY Harbor Healthcare System, New York, NY
[Completed]
New York, New York, United States, 10010
Contact: Mike Hyre, MS 212-951-3339 michael.hyre@VA.gov

United States, Texas
Center for the Intrepid/ Brooke Army Medical Center
[Completed]
San Antonio, Texas, United States, 78234
Contact: Sandra Jarzombek 210-916-8295
Sandra.l.jarzombek.ctr@mail.mil

United States, Florida
James A. Haley Veterans' Hospital, Tampa, FL
[Recruiting]
Tampa, Florida, United States, 33612
Contact: Deborah Dreschnack, PhD 813-588-3946
Deborah.Dreschnack@va.gov

IPDSharing

Plan to Share IPD: No

References

Citations:

Resnik L. Research update: VA study to optimize DEKA arm. J Rehabil Res Dev. 2010;47(3):ix-x. PubMed 20665342

Resnik L, Etter K, Klinger SL, Kambe C. Using virtual reality environment to facilitate training with advanced upper-limb prosthesis. J Rehabil Res Dev. 2011;48(6):707-18. PubMed 21938657

Resnik L, Meucci MR, Lieberman-Klinger S, Fantini C, Kelty DL, Disla R, Sasson N. Advanced upper limb prosthetic devices: implications for upper limb prosthetic rehabilitation. Arch Phys Med Rehabil. 2012 Apr;93(4):710-7. doi: 10.1016/j.apmr.2011.11.010. PubMed 22464092

Resnik L, Latlief G, Klinger SL, Sasson N, Walters LS. Do users want to receive a DEKA Arm and why? Overall findings from the Veterans Affairs Study to optimize the DEKA Arm. Prosthet Orthot Int. 2014 Dec;38(6):456-66. doi: 10.1177/0309364613506914. Epub 2013 Nov 28. PubMed 24286806

Resnik L, Klinger SL, Etter K. The DEKA Arm: its features, functionality, and evolution during the Veterans Affairs Study to optimize the DEKA Arm. Prosthet Orthot Int. 2014 Dec;38(6):492-504. doi: 10.1177/0309364613506913. Epub 2013 Oct 22. PubMed 24150930

Barredo J, Acluche F, Disla R, Fantini C, Fishelis L, Sasson N, Resnik L. Appropriateness of advanced upper limb prosthesis prescription for a patient with cognitive impairment: a case report. Disabil Rehabil Assist Technol. 2017 Aug;12(6):647-656. doi: 10.1080/17483107.2016.1201155. Epub 2016 Jul 19. PubMed 27434169

Resnik L, Borgia M, Acluche F. Brief activity performance measure for upper limb amputees: BAM-ULA. Prosthet Orthot Int. 2017 Jan 1:309364616684196. doi: 10.1177/0309364616684196. [Epub ahead of print] PubMed 28091278

Resnik L, Borgia M, Acluche F. Timed activity performance in persons with upper limb amputation: A preliminary study. J Hand Ther. 2017 May 6. pii: S0894-1130(17)30091-1. doi: 10.1016/j.jht.2017.03.008. [Epub ahead of print] PubMed 28487130

Resnik LJ, Borgia ML, Acluche F. Perceptions of satisfaction, usability and desirability of the DEKA Arm before and after a trial of home use. PLoS One. 2017 Jun 2;12(6):e0178640. doi: 10.1371/journal.pone.0178640. eCollection 2017. PubMed 28575025

Links:

Available IPD/Information:
